# A photoaffinity glycan-labeling approach to investigate immunoglobulin glycan-binding partners

**DOI:** 10.1093/glycob/cwad055

**Published:** 2023-07-27

**Authors:** Miles D Holborough-Kerkvliet, Greta Mucignato, Sam J Moons, Venetia Psomiadou, Rohit S R Konada, Nichole J Pedowitz, Matthew R Pratt, Theresa Kissel, Carolien A M Koeleman, Rayman T N Tjokrodirijo, Petrus A van Veelen, Thomas Huizinga, Karin A J van Schie, Manfred Wuhrer, Jennifer J Kohler, Kimberly M Bonger, Thomas J Boltje, Reinaldus E M Toes

**Affiliations:** Department of Rheumatology, Leiden University Medical Center, Albinusdreef 2, 2333 ZA, Leiden, The Netherlands; Department of Rheumatology, Leiden University Medical Center, Albinusdreef 2, 2333 ZA, Leiden, The Netherlands; Department of Synthetic Organic Chemistry, Radboud University, Toernooiveld 1, Mercator III, 6525 ED, Nijmegen, The Netherlands; Department of Synthetic Organic Chemistry, Radboud University, Toernooiveld 1, Mercator III, 6525 ED, Nijmegen, The Netherlands; Department of Biochemistry, University of Texas Southwestern, 5323 Harry Hines Boulevard, Dallas, TX 75390-09185, United States; Department of Chemistry, University of Southern California, Los Angeles, CA 90089, United States; Department of Chemistry, University of Southern California, Los Angeles, CA 90089, United States; Department of Rheumatology, Leiden University Medical Center, Albinusdreef 2, 2333 ZA, Leiden, The Netherlands; Center for Proteomics and Metabolomics, Leiden University Medical Center, Albinusdreef 2, 2333 ZA, Leiden, The Netherlands; Center for Proteomics and Metabolomics, Leiden University Medical Center, Albinusdreef 2, 2333 ZA, Leiden, The Netherlands; Center for Proteomics and Metabolomics, Leiden University Medical Center, Albinusdreef 2, 2333 ZA, Leiden, The Netherlands; Department of Rheumatology, Leiden University Medical Center, Albinusdreef 2, 2333 ZA, Leiden, The Netherlands; Department of Rheumatology, Leiden University Medical Center, Albinusdreef 2, 2333 ZA, Leiden, The Netherlands; Center for Proteomics and Metabolomics, Leiden University Medical Center, Albinusdreef 2, 2333 ZA, Leiden, The Netherlands; Department of Biochemistry, University of Texas Southwestern, 5323 Harry Hines Boulevard, Dallas, TX 75390-09185, United States; Department of Synthetic Organic Chemistry, Radboud University, Heyendaalseweg 135, 6525 AJ, Nijmegen, The Netherlands; Department of Synthetic Organic Chemistry, Radboud University, Toernooiveld 1, Mercator III, 6525 ED, Nijmegen, The Netherlands; Department of Rheumatology, Leiden University Medical Center, Albinusdreef 2, 2333 ZA, Leiden, The Netherlands

**Keywords:** antibodies, B-cell receptor, CD22, photoaffinity labeling, variable domain glycans

## Abstract

Glycans play a pivotal role in biology. However, because of the low-affinity of glycan-protein interactions, many interaction pairs remain unknown. Two important glycoproteins involved in B-cell biology are the B-cell receptor and its secreted counterpart, antibodies. It has been indicated that glycans expressed by these B-cell-specific molecules can modulate immune activation via glycan-binding proteins. In several autoimmune diseases, an increased prevalence of variable domain glycosylation of IgG autoantibodies has been observed. Especially, the hallmarking autoantibodies in rheumatoid arthritis, anti-citrullinated protein antibodies, carry a substantial amount of variable domain glycans. The variable domain glycans expressed by these autoantibodies are N-linked, complex-type, and α2–6 sialylated, and B-cell receptors carrying variable domain glycans have been hypothesized to promote selection of autoreactive B cells via interactions with glycan-binding proteins. Here, we use the anti-citrullinated protein antibody response as a prototype to study potential in solution and in situ B-cell receptor–variable domain glycan interactors. We employed SiaDAz, a UV-activatable sialic acid analog carrying a diazirine moiety that can form covalent bonds with proximal glycan-binding proteins. We show, using oligosaccharide engineering, that SiaDAz can be readily incorporated into variable domain glycans of both antibodies and B-cell receptors. Our data show that antibody variable domain glycans are able to interact with inhibitory receptor, CD22. Interestingly, although we did not detect this interaction on the cell surface, we captured CD79 β glycan–B-cell receptor interactions. These results show the utility of combining photoaffinity labeling and oligosaccharide engineering for identifying antibody and B-cell receptor interactions and indicate that variable domain glycans appear not to be lectin *cis* ligands in our tested conditions.

## Introduction

The glycosylation of proteins (i.e. glycoproteins) is an ubiquitous phenomenon in eukaryotes. Glycans can modulate many properties of the proteins that carry them, including but not limited to protein folding, steric hindrance, degradation, adhesion, and signaling (Moremen et al. 2012). The role of glycans in human physiology has become an important field of study and touches many of its important facets, of which the immune system is a key example (Maverakis et al. 2015). An important immunological process that is in part mediated by glycan interactions is the regulation of B cells via the membrane-bound B-cell receptor (BCR). Both BCRs and antibodies (Abs), their secreted counterparts, are prominent glycoproteins and can express isotype- and subclass-dependent, conserved and non-conserved N-linked glycans (Epp et al. 2016; van de Bovenkamp et al. 2016). Upon antigen binding to the BCR, an intracellular signaling cascade is initiated that results in the activation, proliferation, and differentiation of the B cell (Rajewsky 1996), whereas secreted Abs are able to neutralize antigen and recruit effector functions (Chiu et al. 2019). Moreover, the activation of the B cell can be modulated by various co-receptors and soluble receptors in a spatiotemporal manner that can directly affect B-cell fate (Nitschke 2005; Muller et al. 2013; Giovannone et al. 2018; Meyer et al. 2018; Adachi et al. 2012). Some of these BCR co-receptors, such as CD22 (SIGLEC-2), a member of the sialic acid-binding, immunoglobulin-type lectin (SIGLEC) family of proteins, are able to bind sialic acids via their extracellular domain (Han et al. 2005). Differential expression of sialic acids on *cis* ligands regulates the distribution of CD22 and its proximity to the BCR, and is able to inhibit B-cell activation via these mechanisms (Muller et al. 2013). Additionally, it is reported that glycans on IgM-BCRs and Abs might directly interact with CD22 in *cis* (Alborzian Deh Sheikh et al. 2018; Peaker and Neuberger 1993) and *trans* (Adachi et al. 2012; i.e. interactions on the same cell, or opposing cells), respectively. However, it is less clear if these findings are a result of technical limitations, as other studies could not substantiate these BCR–CD22 interactions (Han et al. 2005; Zhang and Varki 2004; Muller et al. 2013). These issues highlight one of the key difficulties in identifying glycan interactions: studying glycan interactions in situ is inherently challenging, because of their relatively low affinities, noncovalent interactions, and fast dissociation rates (Paulson et al. 2006; Yu et al. 2012; Tanaka and Kohler 2008). In practical terms, this means that low affinity glycan interactions might not be picked up by immunoprecipitation or that the scrambling of glycan–ligand interactions that may occur in lysates could result in the detection of interactions that do not occur in situ. More stringent and robust methods to study glycan interactions have come from the fields of chemical biology and chemical proteomics. Methods that are able to directly label glycan-binding partners in in situ contexts, such as photoaffinity labeling (PAL), are one of such methods and have been used in elucidating various novel glycan interactions (Han et al. 2005; Ramya et al. 2010; Tanaka and Kohler 2008). To elucidate glycan interactions with PAL, carbohydrate analog functionalized with UV-activatable moieties such as diazirines or aryl azides are used. Upon UV irradiation, these molecules produce reactive carbenes that can form covalent interactions with proximal-binding partners (Yu et al. 2012). Subsequently, these in situ formed, covalently linked, glycan–ligand complexes can then be isolated and studied using conventional techniques such as western blotting or mass spectrometry. The required carbohydrate analog can be introduced by metabolic oligosaccharide engineering or by exo-enzymatic engineering (Dube and Bertozzi 2003; Yarravarapu et al. 2022; Babulic and Capicciotti 2022). Metabolic oligosaccharide engineering relies on the endogenous metabolic pathways in cells for the uptake, processing, and incorporation of the respective carbohydrate analog. Conversely, exo-enzymatic engineering utilizes the capacity of recombinant, exogenous enzymes to append the respective carbohydrates to the cell surface glycans (Babulic and Capicciotti 2022; Yarravarapu et al. 2022). Inspired by these techniques, we aimed to study the interactions mediated by BCR and Abs glycans. In particular, the B-cell responses in B-cell-mediated autoimmune diseases are of interest, as in various autoimmune diseases the disease-specific autoantibodies are enriched in non-conserved variable domain glycans (VDGs; Hafkenscheid et al. 2017, 2019; Vergroesen et al. 2018; Kempers et al. 2018; Koers et al. 2023). These VDGs have been shown to be introduced via somatic hypermutation (Vergroesen et al. 2018) and are N-linked, complex-type, and α2–6 sialylated glycans (Rombouts et al. 2016; Kempers et al. 2018). These sialylated VDGs expressed on the autoimmune disease-specific BCRs have been suggested to interact in *cis* with sialic acid-binding molecules on the B-cell surface and fine-tune B-cell signaling to favor survival of autoreactive B-cell clones (van de Bovenkamp et al. 2016; Koers et al. 2023; Vergroesen et al. 2018; Kissel et al. 2023; Supplementary Fig. 1). In the context of rheumatoid arthritis (RA), levels of autoantibody variable domain glycosylation have been shown to rise in conjunction with disease onset (Hafkenscheid et al. 2019; Kissel et al. 2019) and are ubiquitously expressed at high levels in established RA (Rombouts et al. 2016; Kempers et al. 2018). Therefore, we focused our attention on the VDGs of the RA-specific, anti-citrullinated protein antibody (ACPA) response. To investigate potential *cis* interactions of ACPA BCR VDGs and sialic acid-binding proteins, we employed exo-enzymatic engineering using recombinant α2–6 sialyltransferases (ST6GAL) to generate SiaDAz-carrying ACPA monoclonal antibodies (mAbs) and used metabolic oligosaccharide engineering to introduce SiaDAz into BCRs. We show with LC–MS, in an Ab and BCR-specific manner that SiaDAz is readily incorporated into respective VDGs. Moreover, we show that Abs functionalized with SiaDAz can be used to screen potential lectin-binding partners in a highly specific manner as Abs could be UV- and SiaDAz-dependently be cross-linked to *Sambucus Nigra* Agglutinin (SNA) and CD22 (α2–6-Sia-binding lectins), but not to SIGLEC-1 (α2–3-Sia-binding lectin) or a range of non-lectin proteins. Finally, although we show the non-perturbing nature of metabolic oligosaccharide engineering, the recapitulation of UV- and SiaDAz-dependent CD22-multimerization, and the UV-PAL functionality of isolated BCRs, in situ we do not observe VDG-binding partners, including CD22. Interestingly, we detected VDG-independent cross-linking of CD79 β to the BCR heavy chain, which likely interact because of their close proximity. Together, our data indicate that the BCR VDGs do not seem to be involved in *cis* interactions in situ but underscores the robust nature of PAL for detecting novel glycan interactions such as the CD79 β–BCR interaction we observed.

## Results

### Exo-enzymatic engineering of ACPA mAbs with UV-photo activatable SiaDAz

**Fig. 1:**
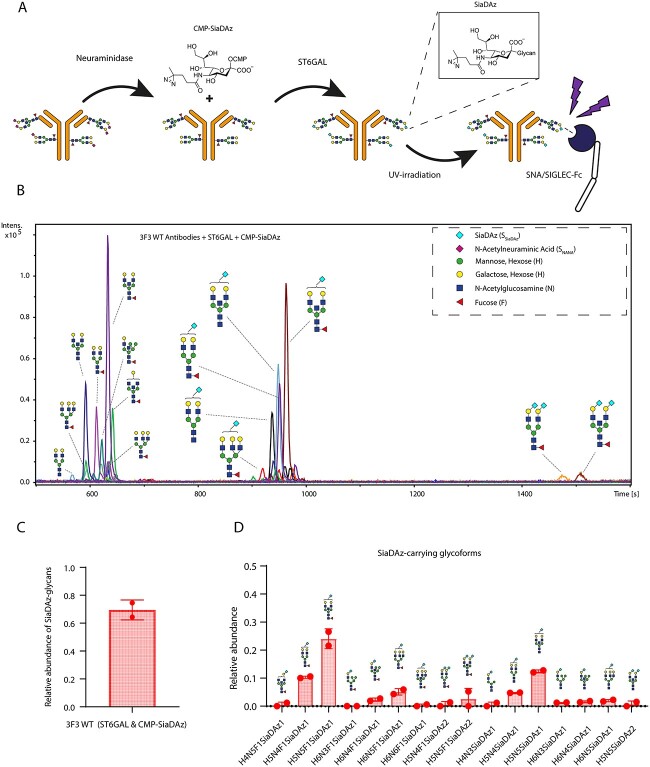
a) Graphical depiction of our exo-enzymatic engineering approach: mAbs produced from an RA patient-derived BCR sequence were treated with neuraminidase to remove sialic acids. CMP-SiaDAz was enzymatically appended onto the terminal galactoses on the fc and VDGs by α2–6 sialyltransferase. mAbs functionalized with SiaDAz are able to form UV-induced covalent interactions with proximal glycan-binding partners. All canonical monosaccharide symbols in this manuscript follow the symbol nomenclature for glycans (SNFG) system (Varki et al. 2015). The non-canonical, synthetic sialic acid, SiaDAz is annotated as a cyan diamond. In the text, we refer to N-acetylneuraminic acid and SiaDAz as S_NANA_ and S_SiaDAz_, respectively. b) Extracted ion chromatograms of enzymatically released Fc and variable domain complex and hybrid-type glycans of an exo-enzymatically SiaDAzylated ACPA that passed quality control parameters. c) Relative abundances of all detected complex and hybrid-type glycans that carry a SiaDAz-molecule. *N* = 2 experimental replicates. d) Relative abundances of all detected complex and hybrid-type glycoforms that carry a SiaDAz-molecule. *N* = 2 experimental replicates.

To identify potential-binding partners of VDGs in solution, we generated ACPA IgG mAbs expressing VDGs from an RA patient-derived BCR sequence (from here on referred to as 3F3 wild type (WT); Kissel et al. 2020, 2022; Supplementary Fig. 2a). This particular 3F3 WT clone expressed a total of 6 VDGs (2 VDGs per V_H_ and 1 VDG per V_L_) in addition to the 2 conserved Fc glycans. Because of the technical challenges that come with studying in situ glycan interactions, we decided to employ CMP-SiaDAz, a synthetic UV-activatable N-Acetylneuraminic acid (NANA) analog that has the capability of covalently capturing glycan interactions (Dube and Bertozzi 2003; Yarravarapu et al. 2022; Babulic and Capicciotti 2022; Tanaka and Kohler 2008). CMP-SiaDAz is obtained after chemoenzymatic conversion of SiaDAz and can be enzymatically incorporated into terminally galactosylated glycans by sialyltransferase activity (Yarravarapu et al. 2022; Fig. 1a). Abs were treated with sialidase to remove the terminally located NANA (Fig. 1a and Supplementary Fig. 2b) and subsequently incubated with α2–6 sialyltransferase and CMP-SiaDAz (Fig. 1a). To assess the glycosylation of these exo-enzymatically modified mAbs, Fc and VDGs were enzymatically released and analyzed with LC–MS (Fig. 1b). Quantification of complex and hybrid-type glycans was performed on the glycans that passed data analysis quality control parameters (see Materials and Methods). To distinguish between NANA and SiaDAz in the glycan nomenclature, we will from here on refer to them as S_NANA_ or S_SiaDAz_, respectively, and visually depict SiaDAz as a cyan diamond (Fig. 1b). The percentage of total glycans that contained SiaDAz was ~70% (Fig. 1c). SiaDAz was observed in a wide range of glycoforms, of which H5N5F1S_SiaDAz_1 was the most abundant (Fig. 1d and Supplementary Fig. 2c). As total glycan release experiments cannot determine the respective Fc or variable domain origins of the glycans, we decided to assess the exo-enzymatic engineering of a variant of the 3F3 WT mAb that expresses only Fc glycans and not VDGs (from here on referred to as 3F3 non-glycosylated (NG)). Similarly for the 3F3 WT, the 3F3 NG glycosylation was assessed by LC–MS (Supplementary Fig. 3a). 3F3 NG was treated with sialidase to remove the terminally located NANA (Supplementary Fig. 3b) and exogenously SiaDAzylated with α2–6 sialyltransferase (Supplementary Fig. 3c and d). The percentage of SiaDAz-carrying glycans in the 3F3 NG was slightly lower (46%; Supplementary Fig. 3d) than for the 3F3 WT (~70%), and SiaDAz was mainly incorporated into the diantennary H5N4F1S_SiaDAz_1 glycan, whereas the predominant SiaDAz-glycan in the 3F3 WT was the diantennary, bisected H5N5F1S_SiaDAz_1 (Fig. 1b and d and Supplementary Fig. 2c). Taking these data into account, as well as the fact that there are only 2 Fc glycans expressed on 3F3 NG versus 8 glycans (2 Fc glycans and 6 VDGs) expressed on 3F3 WT, we suggest that exo-enzymatic engineering leads to a substantial incorporation of SiaDAz in both Fc and VDGs, but that SiaDAz is preferentially exo-enzymatically incorporated into the VDGs.

### Screening for potential-binding glycan partners with SiaDAzylated ACPA IgG mAbs

**Fig. 2:**
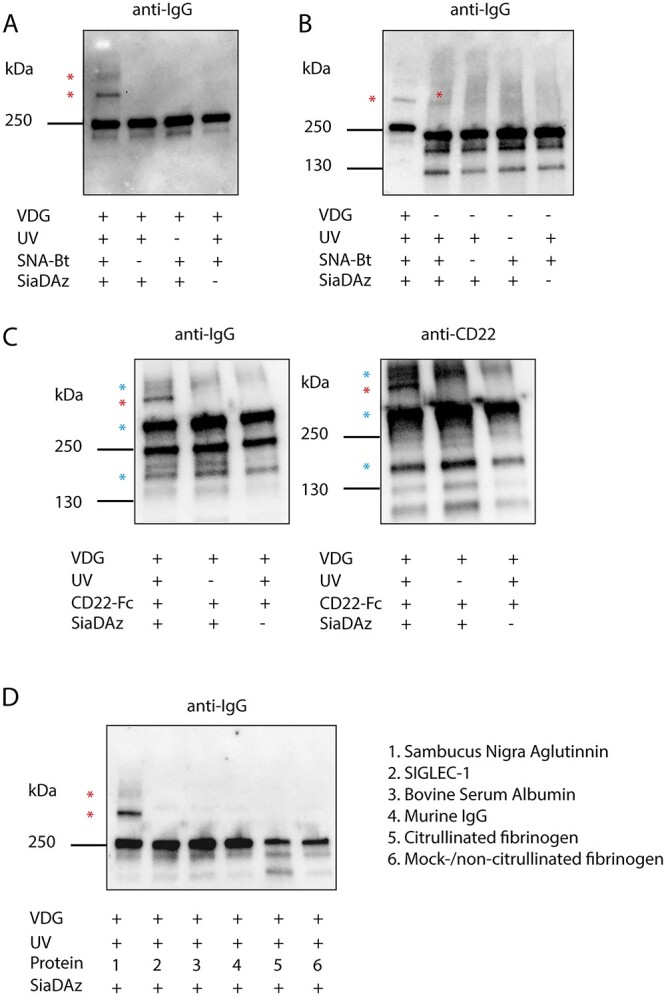
a) Anti-IgG western blot of 3F3 WT (VDG+) mAbs co-incubated with SNA. The presence of UV-, SiaDAz-, and SNA-dependent higher molecular weight bands can be observed. Cross-linked complexes are annotated with a red asterisk. b) Anti-IgG western blot of 3F3 WT and NG (VDG−) mAbs co-incubated with SNA. The lower abundance of UV-, SiaDAz-, and SNA-dependent higher molecular weight bands of 3F3 NG compared with 3F3 WT can be observed. Cross-linked complexes are annotated with a red asterisk. c) Anti-IgG and anti-CD22 western blot of 3F3 WT mAbs co-incubated with CD22-fc. The presence of UV-, SiaDAz-, and SNA-dependent higher molecular weight bands. Cross-linked complexes are annotated with a red asterisk. Blue asterisks indicate the presence of (UV-independent) mono-, di-, and multivalent CD22-fc molecules. d) Anti-IgG western blot comparing the UV- and SiaDAz-dependent PAL capabilities of 3F3 WT of various co-incubated proteins: (1) SNA, (2) SIGLEC-1, (3) bovine serum albumin, (4) murine IgG, (5) citrullinated-fibrinogen, (6) mock-/non-citrullinated-fibrinogen. For each lane, mAbs and proteins were incubated in a 1:2 molar ratio, respectively.

To assess the UV-PAL capabilities of the exo-enzymatically SiaDAzylated mAbs in solution, we co-incubated the SiaDAzylated 3F3 WT mAbs with SNA, a plant-derived lectin known for its specificity for NANA (α2–6) Gal/GalNAc (Shibuya et al. 1987). As evidenced by anti-IgG western blot, UV-exposure induced the presence of higher molecular weight bands that were dependent on the presence of SiaDAz (Fig. 2a). These higher molecular weight bands indicate the presence of the formation of covalent mAb-SNA complexes. To assess the contribution of Fc glycans to the UV-induced cross-linking of mAbs and SNA, we co-incubated 3F3 NG with SNA. Again, higher molecular weight bands were observed on anti-IgG western blot in lanes containing co-irradiated exo-enzymatically SiaDAzylated 3F3 NG and SNA, albeit to a lesser extent than 3F3 WT (Fig. 2b). This could be because of the lower relative incorporation of SiaDAz present in the 3F3 NG (Supplementary Fig. 3c and d), the lower absolute number of glycans present in the Fc compared with the variable domain in the 3F3 WT clone or the lower accessibility of Fc glycans to SNA (Stadlmann et al. 2009; Guhr et al. 2011). After validating the proof of concept by covalently capturing the binding of SNA to Ab glycans, we decided to investigate if SiaDAzylated Abs could be cross-linked to a potentially relevant cell surface sialic acid-binding protein. We focused our attention to CD22 (SIGLEC-2), a B-cell restricted member of the SIGLECs (Nitschke 2005; Meyer et al. 2018). CD22 is an inhibitory BCR co-receptor that has been shown to bind α2–6 sialic acids on various *cis* and *trans* glycoproteins such as neighboring CD22 molecules (Han et al. 2005; Meyer et al. 2018). To validate the results described above and to show the ability of SiaDAzylated mAbs to cross-link to human lectins, we co-incubated SiaDAzylated WT ACPA mAbs and respective controls with CD22-Fc and UV-irradiated the samples. We observed SiaDAz- and UV-dependent higher molecular weight bands indicating IgG-CD22-Fc complexes in both anti-IgG and anti-CD22 blots (Fig. 2c).

To further assess the specificity of the UV PAL capacity of our SiaDAzylated mAbs, we co-incubated them with SNA, SIGLEC-1, murine IgG, bovine serum albumin, citrullinated fibrinogen, and mock/non-citrullinated fibrinogen (Fig. 2d). No higher molecular weight bands were observed in these lanes, including those containing SIGLEC-1 and citrullinated fibrinogen. SIGLEC-1 interacts specifically with α2–3-linked sialoglycans (Shibuya et al. 1987), whereas citrullinated fibrinogen is a cognate citrullinated antigen recognized by the 3F3 ACPA clone, through epitope–paratope interactions (Kissel et al. 2020, 2022). These data taken together show that SiaDAzylated ACPA mAbs can be used to detect potential glycan-binding partners in solution with high specificity by UV-cross-linking them to their respective-binding partners.

### Assessing incorporation of SiaDAz into IgG-BCR glycans

**Fig. 3:**
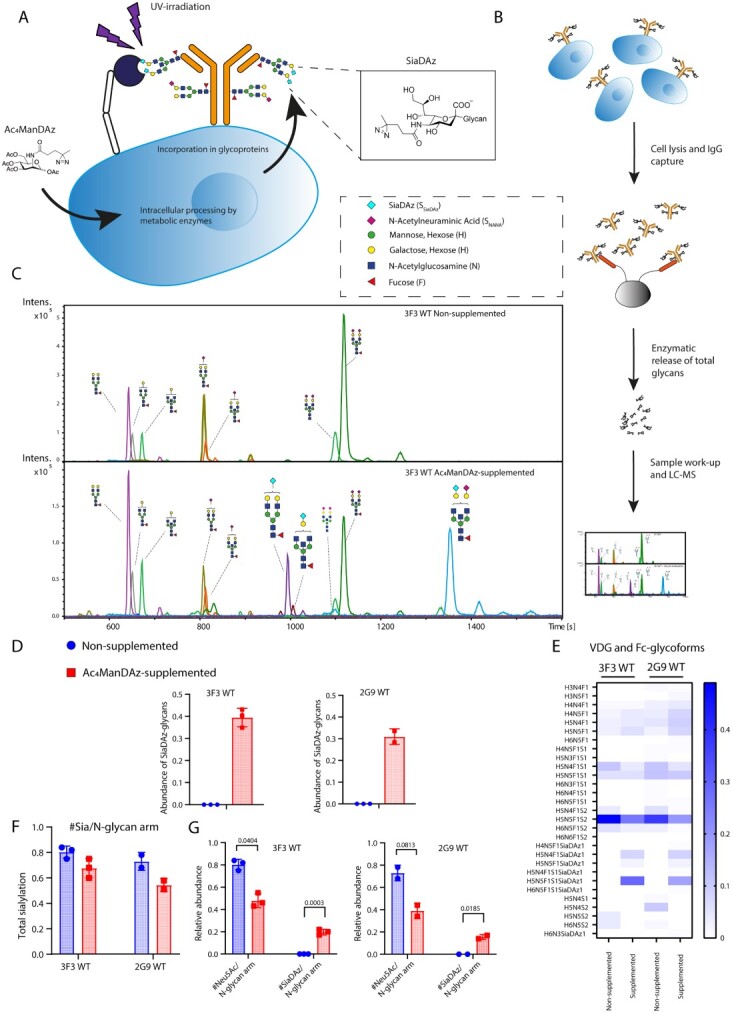
a) Graphical depiction of metabolic oligosaccharide engineering using SiaDAz precursor, Ac_4_MaNDAz in combination with ACPA BCR-expressing B-cell lines. Ac_4_MaNDAz is taken up by B-cell lines via passive diffusion, intracellularly processed by metabolic enzymes and finally incorporated into glycoproteins in the form of SiaDAz. b) Simplified graphical depiction of the protocol for analyzing BCR-specific glycans (full protocol is described in materials and methods). B-cell lines are cultured with or without Ac_4_MaNDAz and lysed. The IgG-BCR is captured using fc-directed affinity matrices. Fc and VDGs are released enzymatically before glycolabeling and purification. Finally, the samples are analyzed by liquid chromatography-mass spectrometry. c) Extracted ion chromatograms of released glycans obtained from 3F3 WT BCRs cultured with or without Ac_4_MaNDAz. d) Elative abundances of SiaDAz-carrying glycans detected in BCR samples cultured with or without Ac_4_MaNDAz for the 3F3 WT and 2G9 WT BCR clones. *N* = 3 biological replicates for 3F3 WT and *n* = 2 biological replicates for 2G9 WT. e) Heat map of the relative abundances of the detected glycans in BCR samples cultured with or without Ac_4_MaNDAz for the 3F3 WT and 2G9 WT BCR clones. *N* = 3 biological replicates for 3F3 WT and *n* = 2 biological replicates for 2G9 WT. f) Relative abundances of the total sialylation (percentage of NANA and SiaDAz combined, corrected for the number of glycan arms) of BCR samples cultured with or without Ac_4_MaNDAz for the 3F3 WT and 2G9 WT BCR clones. *N* = 3 biological replicates for 3F3 WT and *n* = 2 biological replicates for 2G9 WT. g) Relative abundances of the sialylation and SiaDAzylation (percentage of respective sialic acid species, corrected for the number of glycan arms) of BCR samples cultured with or without Ac_4_MaNDAz for the 3F3 WT and 2G9 WT BCR clones. *N* = 3 biological replicates for 3F3 WT and *n* = 2 biological replicates for 2G9 WT (*P* < 0.05, unpaired *t*-test with Bonferroni–Dunn correction).

After showing the specificity for ACPA mAbs to form UV-dependent, covalent interactions with sialic acid-binding lectins via their VDGs in solution, we aimed to validate VDG ligands in a cell surface, in situ context using ACPA B cells. To this end, Burkitt’s lymphoma human B-cell lines expressing RA patient-derived citrulline-directed IgG-BCRs carrying VDGs (WT) and BCRs lacking VDGs were generated (NG), as described previously (Kissel et al. 2020, 2022). In addition to the 3F3 WT ACPA clone which expresses a total of 2 V_H_ VDGs and 1 V_L_ VDG, we also included a 2G9 ACPA clone, which expresses a total of 3 V_H_ VDGs and 1 V_L_ VDG. To incorporate SiaDAz into the B-cell glycoproteins, we followed a metabolic oligosaccharide engineering approach (Dube and Bertozzi 2003; Fig. 3a). Ac_4_MaNDAz can be metabolically converted to SiaDAz by enzymes in the sialic acid biosynthetic pathway and be incorporated into sialoglycans (Bond et al. 2011; Pham et al. 2015; Tanaka and Kohler 2008). B cells were cultured with or without Ac_4_MaNDAz supplemented into the medium. Incorporation of SiaDAz was assessed by capturing IgG-BCRs from lysates followed by the enzymatic release of Fc and VDGs and LC–MS (Fig. 3b and Supplementary Fig. 4a and b). The appearance of additional glycan peaks was observed in the chromatograms of released glycans from B cells cultured in the presence of Ac_4_MaNDAz, with mass over charge (*m/z*) values corresponding to glycans carrying either a single SiaDAz-molecule or both a SiaDAz and Neu5Ac-molecule (Fig. 3c and Supplementary Fig. 4b). After quantification of the LC–MS data, SiaDAz was found to be incorporated in 39 and 30% of complex and hybrid type glycans for the 3F3 WT and 2G9 WT clones, respectively (Fig. 3d). Additionally, the main sialoglycoforms that were observed in non-supplemented samples were recapitulated in samples supplemented with Ac_4_MaNDAz (Fig. 3c and e and Supplementary Figs 4 and 5). This is evidenced by the 2 most abundant NANA-containing glycans, H5N5F1S_NANA_2 and H5N4F1S_NANA_1, and their SiaDAz-containing glycan counterparts, H5N5F1S_NANA_1S_SiaDAz_1 and H5N4F1S_SiaDAz_1. A slight drop in the total sialylation (e.g. combined percentage of NANA and SiaDAz, corrected for the number of sialic acids per antennae as a percentage of complex and hybrid-type glycans) was observed for the Ac_4_MaNDAz supplemented conditions from 80 to 68% for 3F3 WT clone and from 73 to 51% for the 2G9 WT clone, respectively (Fig. 3f and g). This reduction could be explained by the variability in the metabolism of unnatural sialic acid analog and their precursors in observed in cell lines, as reported in previous studies (Pham et al. 2015).

The diantennary, bisected and disialylated H5N5F1S_NANA_2 glycan has previously been shown to be predominately expressed in the variable domain and is found to a much lesser extent in the Fc glycans of ACPA Abs (Kempers et al. 2018). After the enzymatic release of glycans, however, the peptide and glycan are uncoupled and thus, information about the location of the respective glycans cannot be determined. In order to detect VDG-dependent interactions with our UV-PAL approach, it is important that SiaDAz is readily incorporated into the VDG. To this end, we assessed the total BCR glycosylation of 3F3 and 2G9 clones that exclusively expressed Fc glycans and lacked variable domain glycosylation (3F3 and 2G9 NG) of cells supplemented with or without Ac_4_MaNDAz. We found that SiaDAz was incorporated into ~30% of Fc glycans for both 3F3 NG and 2G9 NG (Supplementary Figs 6b and 7b). The characteristic VDG H5N5F1S_NANA_2 was not detected in 3F3 NG glycans and was only present in 2G9 NG glycans to a minor extent (Supplementary Figs 6 and 7). The SiaDAz-carrying counterpart, H5N5F1S_NANA_1S_SiaDAz_1 was not detected in either 3F3 NG or 2G9 glycans. The lack of the H5N5F1S_NANA_2 and H5N5F1S_NANA_1S_SiaDAz_1 glycans in the Fc of the NG clones indicates that these glycans are derived from the variable domains in the WT clones. Taken together, these data show robust incorporation of Ac_4_MaNDAz into IgG-BCR glycans, suggest that it is predominately incorporated into VDGs, and show that this incorporation is mainly present in the form of the characteristic H5N5F1S_NANA_1S_SiaDAz_1.

### Assessing cellular perturbations, functionality, and in situ VDG-binding partners

**Fig. 4:**
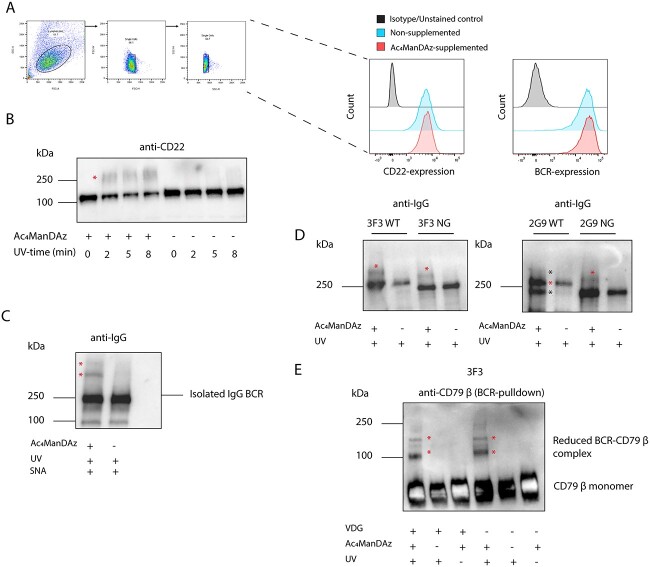
a) Histogram showing the cell surface expression of CD22 and the BCR on cells cultured with or without Ac_4_MaNDAz. Gating strategy included gating on live cells (FSC-A versus SSC-A), followed by doublet discrimination (FSC-H versus FSC-W and SSC-H versus SSC-W). Anti-CD8 APC was used as a CD22-isotype control. b) Anti-CD22 western blot showing the UV- and Ac_4_MaNDAz-dependent induction of CD22-multimers. c) Anti-IgG western blot showing the presence of UV- and Ac_4_MaNDAz-dependent cross-linking of BCRs isolated from Ac_4_MaNDAz-supplemented B cells and SNA. d) Anti-IgG western blot showing VDG-independent but UV- and Ac_4_MaNDAz-dependent higher molecular weight bands above the BCR band. The cross-linked complexes are annotated with a red asterisk. The black asterisks indicate the bands corresponding to the 2G9 WT BCR carrying an unoccupied LC and fully occupied glycan sites, respectively. e) Anti-CD79 β western blot after pulldown of the BCR and reduction of the samples. Only UV- and Ac_4_MaNDAz-dependent CD79 β complexes are present after reduction of the samples and can be found in WT and NG samples.

To ensure that culturing ACPA B cells with Ac_4_MaNDAz and its subsequent incorporation into glycoproteins was not to the detriment of their receptor cell surface expression, we used flow cytometry to assess the expression of the IgG-BCR and CD22 (Fig. 4a). No difference was seen in the expression of the BCR and CD22 between cells cultured with and without Ac_4_MaNDAz, indicating the non-perturbative nature of our Ac_4_MaNDAz metabolic oligosaccharide engineering approach. Next, we aimed to validate the functionality of the incorporated SiaDAz in an in situ context. To this end, we recapitulated the sialic acid-dependent *cis*-multimerization of CD22, a known SIGLEC–sialic acid interaction that had previously been elucidated by Han et al. and replicated by others (Han et al. 2005; Tanaka and Kohler 2008). UV-exposure of cells supplemented with Ac_4_MaNDAz clearly induced the formation of multimeric CD22-complexes that were absent in cells cultured without Ac_4_MaNDAz (Fig. 4b). After having validated the approach, we next, directly assessed the PAL capabilities of the BCRs expressing SiaDAzylated glycans. To this end, we isolated IgG-BCRs of cells supplemented with or without Ac_4_MaNDAz and co-incubated them with SNA during UV-irradiation. Similarly to what we observed for ACPA mAbs, Ac_4_MaNDAz-dependent higher molecular weight bands were observed (Fig. 4c). After confirming the lack of perturbation, and the functionality of Ac_4_MaNDAz in these BCR-directed in solution and cellular contexts, we aimed to investigate if we could detect in situ BCR VDG-binding partners. We assessed the BCRs of resting B cells that were cultured with or without Ac_4_MaNDAz and exposed to UV by western blot for higher molecular weight bands. Surprisingly, higher molecular weight bands were detected for both the 3F3 WT, NG and the 2G9 NG, indicating a sialic acid-dependent interaction that was not VDG dependent (Fig. 4d and Supplementary Fig. 8b). The higher molecular bands are less clearly seen in the 2G9 WT clone. The 2G9 WT light chain (LC) glycosylation site has been found to be incompletely occupied (Kissel et al. 2022). This visually separates the non-reduced 2G9 BCR into 2 separate bands (annotated by a black asterisk), which we suspect obscures the UV and Ac_4_ManDAz-induced cross-linked bands.

Moreover, these higher molecular weight bands were also detected in samples of B cells stimulated with Latrunculin-A and Pervanadate, indicating no effect of the B-cell activation state on the presence of cross-linked complexes (Supplementary Fig. 8a). The higher molecular weight bands were analyzed by mass spectrometry. CD79 β was identified by the presence multiple CD79 β-derived peptides (IWQSPR, QEMDENPQLK, WSVGEHPQE, and VMGFSTLAQLK) in the samples exposed to UV and cultured with Ac_4_MaNDAz and not in the negative controls (Table SI). Conversely, CD79 α was only found in some of these bands, and CD22 or any other sialic acid-binding proteins were not detected at all. Anti-CD79 β western blots confirmed the MS findings and detected the presence of CD79 β at the expected mobility (Supplementary Fig. 8c). CD79 β was also present at higher apparent molecular weights in samples that were not exposed to UV or cultured with Ac_4_MaNDAz. We suspect that these captured CD79 β–BCR complexes are the result of the close proximity of CD79 β and the BCR, which are co-localized via strong but noncovalent interactions of CD79 β with the BCR (Schamel and Reth 2000; Su et al. 2022; Ma et al. 2022). Reducing these samples and staining for CD79 β did not affect the presence of the UV- and Ac_4_MaNDAz-dependent bands, whereas these bands disappeared in the control conditions (Fig. 4e). These findings indicate that the noncovalent CD79 β–BCR interactions are covalently captured and stabilized upon UV-irradiation in the presence of Ac_4_MaNDAz. Concluding, these data indicate that BCR VDGs do not interact *in cis* with glycan-binding partners in situ. However, our data indicates that CD79 β does interact with the BCR, conceivably because of its close proximity to the BCR.

## Discussion

In this study, we report the use of metabolic and enzymatic oligosaccharide engineering to introduce the UV-activatable, diazirine-moiety carrying sialic acid analog, SiaDAz, into ACPA IgG mAb and IgG-BCR Fc and VDGs. Using these techniques, we first aimed to screen potential VDG-binding partners using SiaDAzylated mAbs, and subsequently elucidate their relevance as a *cis*-VDG-binding partner on ACPA B cells. We assessed the incorporation rates of SiaDAz in ACPA IgG-mAbs and IgG-BCR glycans by IgG-specific-capturing followed by enzymatic release of glycans and LC–MS analysis. To our knowledge, such a protein-specific and detailed analysis of the incorporation levels of sialic acid analog, such as SiaDAz, and the quantification of the various SiaDAz-carrying glycans has not been reported previously. In exo-enzymatically SiaDAzylated mAbs, we observed the incorporation of SiaDAz in ~70% of all glycans, of which the majority was incorporated into monoSiaDAzylated (S_SiaDAz1_1) glycans. Moreover, to assess the relative contributions of the Fc and VDGs to the incorporation of SiaDAz, we exo-enzymatically SiaDAzylated mAbs lacking VDGs (NG). Quantification of these data suggest that SiaDAz is incorporated into ~46% of Fc glycans, a slightly lower incorporation rate than observed for WT mAbs. Taking into account the higher absolute number of VDGs compared with Fc in this particular mAb clone, we conclude that SiaDAz is preferentially appended to VDGs. Proof of concept UV-PAL experiments with SNA, an α2–6 sialic acid-binding lectin, show that these incorporation levels are sufficient for inducing cross-linking and that they can result in clearly visible bands in western blots. Our data, therefore, suggest that Fc glycans contribute less to cross-linking to binding partners than VDGs, which could result from the lower Fc incorporation of SiaDAz, the absolute lower number of Fc glycans or the steric hindrance of the IgG Fc tail itself, which has been described in the context of the SNA-mediated IgG fractionation (Stadlmann et al. 2009; Guhr et al. 2011). More physiologically relevant, we also show that exo-enzymatically SiaDAzylated WT mAbs are able to form UV-dependent, higher molecular weight complexes with α2–6-linked sialic acid-binding SIGLECs, such as CD22 but not the α2–3-linked sialic acid SIGLEC-1. Binding to proteins that do not have lectin properties was not observed. Even in the case of citrullinated-fibrinogen, which is an antigen of the 3F3 clone and can interact with the 3F3 paratope that is proximally located to the VDGs (Kissel et al. 2020, 2022), we did not observe higher molecular weight complexes. Although we cannot exclude that these interactions fall below the detection limit of the anti-IgG western blot, our data indicate that capturing SiaDAz-dependent interactions requires very close proximity. Such close proximity is likely present during interactions of α2–6 SiaDAzoglycans and α2–6 sialic acid-binding proteins, or in the case of proximally located SiaDAzoglycans and appropriate (hydrophilic) amino acid residues, such as for the cross-linking we observed between CD79 β and the BCR.

To detect in situ ligands of ACPA IgG-BCR VDGs, we employed metabolic oligosaccharide engineering. Metabolic oligosaccharide engineering leads to a robust incorporation of SiaDAz as the abundance of SiaDAz in ACPA IgG-BCR glycans reached ~30 and 40% in the 3F3 WT and 2G9 WT clone, respectively. BCR-specific total glycan release experiments show that SiaDAz was mainly incorporated in the di-sialylated, bisecting GlcNAc-carrying H5N5F1S_NANA_1S_SiaDAz_1 glycoform and that compared with non-supplemented cells, the glycoforms were not altered by SiaDAz incorporation. Comparing these SiaDAz incorporation levels and the subsequent SiaDAz-carrying glycoforms to those from the BCR clone counterparts that lack VDGs indicates that SiaDAz is preferentially incorporated in the variable domain, similarly to what we observed for the exo-enzymatically engineered mAbs. Although we showed that CD22 and ACPA mAb VDGs are able to interact and SiaDAz is readily incorporated into IgG-BCR VDGs, we were not able to detect binding of VDGs to CD22 or other proteins in situ in our ACPA B-cell lines. Thus, our data suggest the absence of a *cis* interaction between IgG-BCR VDGs and B-cell surface glycan-binding partners.

These finding might come unexpected, as CD22 and the BCR are able to co-localize in resting and activated IgM B cells (Cao et al. 2018; Muller et al. 2013). Moreover, we speculated that CD22, which consists of 7 Ig-like domains (Wilson et al. 1993), and the IgG-BCR, which consists of 4 Ig-like domains (Ma et al. 2022), are ideally positioned to interact as the distally positioned VDGs are mostly located in framework 1, 3 and complementarity-determining regions 1 in the variable domain (Vergroesen et al. 2018). However, it is possible that structural or spatiotemporal limitations of the BCR or CD22 prevent such interactions. Ab initio electron microscopy-based reconstructions of the CD22 ectodomain suggest that CD22 adopts a rigid, rod-like structure that is unchanged upon α2–6 sialyllactose binding (Ereno-Orbea et al. 2017). These data also indicate a length of ~300–312 Å for the ectodomain of CD22 and show that the sialic acid-binding domain is located in the uppermost domain. This might place the IgG-BCR VDGs out of reach of the CD22 sialic acid-binding domain (Ma et al. 2022). In addition, although less likely, it is possible that even though CD22 can come into close proximity of the BCR, it is still too far away for direct glycan interactions to occur. Current PLA and microscopy data suggest that distance between CD22 and the BCR is approximately as close as 10 nm (Muller et al. 2013; Klasener et al. 2014; Cao et al. 2018). This is over 3 times the base of the BCR (Ma et al. 2022) and is possibly too far away for the VDGs to reach. These data taken together are in line with the notion that structural limitations imposed by the cell membrane prevent CD22 from interacting *in cis* with the IgG-BCR VDGs. This would explain other studies that aimed to probe potential *cis* interactions of CD22 and the BCR also failed to show direct glycan interactions, albeit in the context of IgM BCRs (Han et al. 2005; Zhang and Varki 2004; Muller et al. 2013). Moreover, in a recently published study by our group, ACPA B-cell lines expressing VDGs were shown to exhibit differential B-cell biology as evidenced by higher phospho-Syk and Ca^2+^-levels and slower BCR-downmodulation upon stimulation, compared with B-cell lines carrying BCRs lacking VDGs. In CD22 knock-out variants of these cell lines, these phenotypes were maintained, pointing against a causal role of CD22 for this phenotype^31^.

Although, we found no evidence of VDG–glycan-binding protein interactions, we did detect covalently cross-linked CD79 β–BCR complexes. These complexes were detected regardless of the presence of BCR VDGs. We consider it most conceivable that this interaction is explained by the interaction between the BCR and glycans expressed by CD79 b. Recently, the structure of the human IgM BCR has been solved by cryo-EM (Su et al. 2022; Ma et al. 2022). In these studies, it is revealed that the BCR co-receptor, CD79 β, is interlocked with the BCR via noncovalent interactions and that it express glycans that are adjacent to a hydrophilic patch in the BCR heavy chain. Conceivably, these glycans could interact with this patch and further stabilize the BCR complex on the cell surface, which could be investigated by CD79 β glycopeptide analyses and functional assays. Conversely, if it is the case that the BCR Fc glycans are mediating the detected interactions with CD79 β, it would require close proximity of the Fc glycans to CD79 β. It is, at present, unclear whether such interactions between IgG BCR Fc glycans and CD79 β are feasible.

In summary, here we used exo-enzymatic and metabolic oligosaccharide engineering assess potential VDG-binding partners. Initially, we showed that mAbs functionalized with SiaDAz are suitable to covalently and specifically cross-link to α2–6 sialic acid-binding lectins, such as SNA and CD22, and that this driven by variable domain and not Fc glycans. To verify CD22 as an in situ *cis* ligand, B-cell lines expressing patient-derived ACPA BCRs underwent metabolic oligosaccharide engineering. SiaDAz was found to be readily incorporated into BCR VDGs and metabolic oligosaccharide engineering did not negatively impact the cell surface expression of CD22 and the BCR. In addition, we were able to recapitulate the formation of UV-induced CD22 multimers and showed the in-solution UV PAL of isolated BCRs and SNA. No evidence for VDG *cis*-binding partners was observed in western blot or mass spectrometry experiments. In contrast, our data reveal a direct interaction of glycans expressed by CD79 β to the BCR. Lastly, we propose that our methods can be used to elucidate glycan-binding partners of Abs and could additionally be implemented for the detection of BCR VDG *trans* interactions.

## Materials and methods

### Production of mAbs

Detailed description of the complete procedure from the isolation of ACPA B cells from RA patients to the final production of IgG1 Abs in HEK293F cells has been described previously (Kissel et al. 2020, 2022). In short, ACPA B cells were isolated from RA patients using CCP2 and CArgP2 streptavidin-tetramers. ACPA IgG sequences were obtained from single sort B-cell cultures, after validating the CCP2-reactivity of the culture supernatant by ELISA. HEK293F cells (Gibco; Cat. No. R79007) were transfected with pcDNA3.1 expression vectors carrying the ACPA IgG HC/LC variable genes and respective constant domains obtained from UniProt. Ab glyco-engineering was performed by supplementing the cell culture with 500 mM D-galactose and by the additional transfection of glyco-enzyme vectors (GnTIII, ST6galT, and B4GalT1). After 5–6 days, supernatants were harvested, and Abs were purified with CaptureSelect FcXL Affinity Matrix beads (Thermo Fisher; Cat. No. 194328005).

### Ex vivo glyco-engineering of mAbs

To generate ex vivo glyco-engineered mAbs, Abs were first incubated with neuraminidase (Roche; Cat. No 10269611001) overnight at 37 °C. Abs were subsequently purified with CaptureSelect FcXL Affinity Matrix beads and incubated with recombinant human ST6GAL (AA 44-406; Bio Techne; Cat. No. 7620-GT-010) and CMP-SiaDAz or CMP-Neu5Ac for 24 h at 37 °C. The weight ratio of Ab to ST6GAL to substrate was 20/1/20. After 24 h, samples were refrigerated or frozen until further use.

### Chemical synthesis of CMP-SiaDAz

Chemoenzymatic synthesis of CMP-SiaDAz is described in detail in (Yarravarapu et al. 2022). In short, diazirine-modified ManNAc was prepared by chemical synthesis. ManNAc, pyruvate, and CTP were co-incubated with NANA aldolase and CMP-Sialic acid synthetase in the presence of Mg^2+^ and alkaline phosphatase.

### Chemical synthesis of Ac_4_MaNDAz


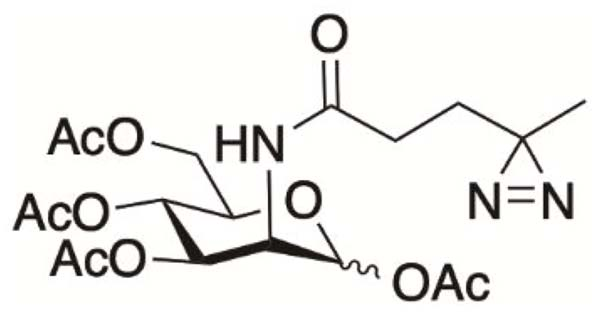


Unprotected MaNDAz was synthesized via 2 ways (e.g. method 1 or 2): initially, Mannosamine HCl (265 mg, 1.23 mmol) was dissolved in MeOH (12.3 ml, 0.1 M). Subsequently, either (i) triethylamine (514 μL, 3 eq.) was added, followed by addition of *N*-Succinimidyl 3-(3-methyl-3*H*-diazirin-3-yl)propanoate (Sun et al. 2017; 415 mg, 1.5 eq.), or (ii) NaOMe dissolved in dry methanol was added dropwise at 0 °C under inert atmosphere and the reaction product was purified using flash chromatography (20:80, MeOH:DCM). After 2 h, the mixture was concentrated, followed by addition of pyridine (4.38 ml, 44 eq.) and acetic anhydride (2.55 ml, 22 eq.), and stirred overnight. The mixture was concentrated, diluted with EtOAc, washed with 0.5 M HCl, water and brine and dried over NaSO_4_ and concentrated. The crude product was purified using flash column chromatography (0–100% EtOAc in heptane) to afford the product (252 mg, 45% over 2 steps). Analytical data correspond to previously reported literature (Tanaka and Kohler 2008).

### Generation of variable domain glycosylated and NG ACPA human B-cell lines

To study the functional effects of VDGs on ACPA B-cell biology, a Ramos human B-cell line lacking genes for IgM and IgD heavy chains, LC, and activation-induced deiminase (AID) was transduced with patient-derived ACPA membrane-bound IgG BCRs. As comparators, WT BCRs expressing VDGs and NG BCRs lacking expression of VDGs were transduced as previously described (Kissel et al. 2020, 2022). The Ramos IgM, IgD, LC, and AID knock-outs (MDL-KO) were kindly provided by Prof. Dr Michael Reth.

### Cell culture and metabolic oligosaccharide engineering

Ramos B cells were cultured in RPMI 1640 (Thermo Fisher; Cat. No. 61870044) containing 8% heat-inactivated FCS, 100 U/ml of Penicillin–Streptomycin (Gibco; Cat. No. 15140-163), 2 mM GlutaMAX (Thermo Fisher; Cat. No 35050038), and 10 mM HEPES (Thermo Fisher; Cat. No. 15630080). To supplement cells with Ac_4_MaNDAz, 16 μl of a 25 mM Ac_4_MaNDAz stock dissolved in EtOH was added to empty wells in a 6-well culture plate. The EtOH was allowed to evaporate, and 2E6 Ramos cells in 4 ml culture medium were added to the respective wells. Cells were incubated for 72 h at 37 °C and 5% CO_2_.

### BCR isolation

B-cell lysates for western blotting were produced by incubating cells in 1% Triton-X in PBS at 37 °C for 1 h. BCRs were captured by incubating the lysates with CaptureSelect FcXL Affinity Matrix beads (Cat. No. 194328010) overnight at 4 °C. For total glycan release experiments, BCRs were released from the affinity matrix in the presence of 100 mM formic acid (FA; pH 2.5) and eluted from polypropylene 96-well filter plates (Orochem; Cat. No. OF 1100). For western blotting experiments, FcXL beads and captured BCRs were boiled in Laemmli buffer (×4; Bio-Rad; Cat. No. 1610747) at 95 °C for 5–10 min and loaded on precast Bio-Rad Mini-PROTEAN TGX gels (4–15%; Bio-Rad; Cat. No. 4561084) in TGS buffer (Bio-Rad; Cat. No. 1610772).

### BCR and Ab glycan analysis

BCRs were isolated and eluted as described above. Eluted BCRs were vacuum dried for 2–3 h at 50–60 °C. Next, BCRs were reconstituted in 10 μl milliQ and 20 μl in 2% Sodium dodecyl sulfate (Merck) for 10 min at 60 °C. A glycan release mixture of 10 μl NP-40 (4%), 10 μl PBS and 1 μl PNGAseF (Sigma-Aldrich; Cat. No. 11365177001) was added to each sample. Samples were shaken horizontally for 5 min at 1,000 rpm. Samples containing glycans and release mixture were incubated overnight at 37 °C. Released glycans were labeled by adding 25 μl of 2-aminobenzoic acid (48 mg/ml in 85% DMSO and 15% glacial acetic acid) and 25 μl of 2-picoline borane (107 mg/ml in DMSO) to the glycans and were incubated for 2 h at 60 °C. Lastly, to purify the labeled glycans, hydrophilic interaction liquid chromatography (HILIC). To this end, 3 mm cotton tips were prepared and loaded into 20 μl pipet tips. Pipet tips were equilibrated by pipetting and discarding 15 μl of milliQ 3 times. Released and labeled glycan samples were brought up to 85% in room temperature acetonitrile (ACN). Released glycans were loaded on the milliQ-equilibrated pipet tips by pipetting up and down 3 times in the 85%-ACN samples. The samples were then washed in 85% ACN, 1% TFA, and liquid was discarded from the pipet tips by pipetting up and down 3 times. Next, the samples were washed in similar manner in 85% ACN and lastly, the glycans were eluted in 10 μl milliQ by pipetting up and down 5 times.

Ex vivo glyco-engineered Abs were loaded on SDS-Page to separate them from the ST6GAL used for glyco-engineering. Instant Blue (Expedeon/Westburg; Cat. No. ISB1L) was incubated with the SDS-Page gels for 15 min to visualize the IgG bands. Bands were cut out and processed individually. Gel pieces were washed twice with washing buffer (25 mM ammonium bicarbonate in milliQ) and subsequently washed twice with 100% ACN. Gel pieces were incubated at 56 °C in reduction buffer (10 mM DTT in washing buffer). Next, gel pieces were washed twice in 100% ACN and reactive cysteines were blocked by incubation with alkylation buffer (55 mM iodoacetamide in washing buffer) for 20 min in the dark. Gel pieces were washed twice with washing buffer and one last time with 100% ACN. After this step, samples were completely destained from Instant Blue and vacuum centrifuged for 5 min at 50–60 °C. To release N-linked glycans, 60 μl of glycan release mixture was added to the gel pieces and incubated overnight at 37 °C. From this step onwards, the same glycan-labeling and HILIC protocols were performed as mentioned above.

### LC–MS protocol

#### Released glycan analysis

Sample (preparation mentioned above) was injected into an Ultimate 3000 RSLCnano system (Thermo Scientific, Breda, the Netherlands) coupled to a quadrupole-TOF-MS (MaXis HD; Bruker Daltonics, Bremen, Germany). The LC system was equipped with an Acclaim PepMap 100 trap column (particle size 5 μm, pore size 100 Å, 100 μm × 20 mm, Thermo Scientific) and an Acclaim PepMap C18 nano analytical column (particle size 2 μm, pore size 100 Å, 75 μm × 150 mm, Thermo Scientific). The samples were loaded and washed on the trap column for 2 min at 15 μl/min with 0.1% FA in water. A mixture of solvent A (0.1% FA in water) and solvent B (95% acetonitrile, 0.1% FA) was applied with a constant flow of 0.7 μL/min using a linear gradient: t(min) = 0, %B = 1; t = 5, %B = 1; t = 30, %B = 50; with washing and equilibration starting at t = 31, %B = 70; t = 35, %B = 70; t = 36, %B = 1; t = 70, %B = 1. The sample was ionized in positive-ion mode using a CaptiveSprayer (Bruker Daltonics) electrospray source at 1250 V. A nanoBooster (Bruker Daltonics) was used to enrich the nitrogen gas with acetonitrile to enhance ionization efficiency (0.2 bar). Mass spectra were acquired with a frequency of 1 Hz and the MS ion detection window was set at mass-to-charge ratio (*m/z*) 550–1,800.

#### Analysis of gel bands

Gel bands were washed, and reduced and alkylated with dithiothreitol and iodoacetamide, respectively, followed by in-gel trypsin digestion using a Proteineer DP digestion robot (Bruker). Peptides were extracted from the gel slices, lyophilized, dissolved in 0.1% FA and subsequently analyzed by on-line C18 nanoHPLC MS/MS with a system consisting of an Easy nLC 1000 gradient HPLC system (Thermo, Bremen, Germany), and a LUMOS mass spectrometer (Thermo). Samples were injected onto a homemade precolumn (100 μm × 15 mm; Reprosil-Pur C18-AQ 3 μm, Dr Maisch, Ammerbuch, Germany) and eluted via a homemade analytical nano-HPLC column (30 cm × 50 μm; Reprosil-Pur C18-AQ 3 um). The gradient was run from 2 to 40% solvent B (20/80/0.1 water/acetonitrile/FA v/v) in 30 min. The nano-HPLC column was drawn to a tip of ∼5 μm and acted as the electrospray needle of the MS source. The LUMOS mass spectrometer was operated in data-dependent MS/MS mode for a cycle time of 3 s, with a HCD collision energy at 32 V and recording of the MS2 spectrum in the orbitrap. MS1 resolution was 120,000, the scan range 350–1,600, at an AGC target of 400,000 with a maximum fill time of 50 ms. Dynamic exclusion after *n* = 1 with an exclusion duration of 10 s. Charge states 2–5 were included. For MS2, precursors were isolated with the quadrupole with an isolation width of 1.2 Da. First mass was set to 110 Da. The MS2 scan resolution was 30,000 with an AGC target of 50,000 @maximum fill time of 60 ms.

In a post-analysis process, raw data were first converted to peak lists using Proteome Discoverer version 2.5 (Thermo Electron), and submitted to the Uniprot *Homo sapiens* minimal database (20,205 entries). For protein identification, Mascot v. 2.2.07 (Matrix Science) was used. Mascot searches were done with 10 ppm and 0.02 Da deviation for precursor and fragment mass, respectively. The enzyme trypsin was specified and up to 2 missed cleavages were allowed. Methionine oxidation and acetylation on the protein N-terminus were set as a variable modification; carbamidomethyl on Cys was set as a fixed modification. Peptides with an FDR < 1% were accepted.

#### LC–MS data processing

The initial visual and manual analysis of the LC–MS data was in Compass DataAnalysis (Bruker Daltonik GmbH, Version 5.0). To quantify the detected glycan peaks, LacyTools v1.1.0-alpha was used (Jansen et al. 2016). Shortly explained, the .raw LC–MS files were converted to mzXML files by using MSConvert (http://proteowizard.sourceforge.io). A file of predicted complex- and hybrid-type glycans was compiled and used for input (nomenclature: Aa = 2-Aminobenzoic Acid, F = Fucose, H = Mannose or galactose, N = N-Acetylglucosamine, S = N-Acetyl Neuraminic Acid and Sdz = SiaDAz) and consisted of the following glycans: *aa1H3N3F1, aa1H3N4F1, aa1H3N5F1, aa1H3N6F1, aa1H4N3F1, aa1H4N4F1, aa1H4N5F1, aa1H5N3F1, aa1H5N4F1, aa1H5N5F1, aa1H6N3F1, aa1H6N4F1, aa1H6N5F1, aa1H6N6F1, aa1H4N3F1S1, aa1H4N4F1S1, aa1H4N5F1S1, aa1H5N3F1S1, aa1H5N4F1S1, aa1H5N5F1S1, aa1H6N3F1S1, aa1H6N4F1S1, a1H6N5F1S1, aa1H6N6F1S1, aa1H5N4F1S2, aa1H5N5F1S2, aa1H6N4F1S2, aa1H6N5F1S2, aa1H6N6F1S2, aa1H4N3F1sdz1, aa1H4N4F1sdz1, aa1H4N5F1sdz1, aa1H5N3F1sdz1, aa1H5N4F1sdz1, aa1H5N5F1sdz1, aa1H6N3F1sdz1, aa1H6N4F1sdz1, aa1H6N5F1sdz1, aa1H6N6F1sdz1, aa1H5N4F1S1sdz1, aa1H5N5F1S1sdz1, aa1H6N4F1S1sdz1, aa1H6N5F1S1sdz1, aa1H6N6F1S1sdz1, aa1H5N4F1sdz2, aa1H5N5F1sdz2, aa1H6N4F1sdz2, aa1H6N5F1sdz2, aa1H6N6F1sdz2, aa1H3N3, aa1H3N4, aa1H3N5, aa1H3N6, aa1H4N3, aa1H4N4, aa1H4N5, aa1H5N3, aa1H5N4, aa1H5N5, aa1H6N3, aa1H6N4, aa1H6N5, aa1H6N6, aa1H4N3S1, aa1H4N4S1, aa1H4N5S1, aa1H5N3S1, aa1H5N4S1, aa1H5N5S1, aa1H6N3S1, aa1H6N4S1, aa1H6N5S1, aa1H6N6S1, aa1H5N4S2, aa1H5N5S2, aa1H6N4S2, aa1H6N5S2, aa1H6N6S2, aa1H4N3sdz1, aa1H4N4sdz1, aa1H4N5sdz1, aa1H5N3sdz1, aa1H5N4sdz1, aa1H5N5sdz1, aa1H6N3sdz1, aa1H6N4sdz1, aa1H6N5sdz1, aa1H6N6sdz1, aa1H5N4S1sdz1, aa1H5N5S1sdz1, aa1H6N4S1sdz1, aa1H6N5S1sdz1, aa1H6N6S1sdz1, aa1H5N4sdz2, aa1H5N5sdz2, aa1H6N4sdz2, aa1H6N5sdz2, aa1H6N6sdz2*. An alignment file containing abundant glycan peaks and their respective retention times (in seconds) was used to align the liquid chromatography chromatograms. The quality control parameters applied to the LacyTools Excel output file consisted of excluding all glycans that showed a signal-to-noise ratio lower than 9, showed a mass accuracy of −20 ≥ × ≥ 20 for BCR glycan analysis or a mass accuracy of −25 ≥ × ≥ 25 for mAb glycan analysis and an isotopic pattern quality ≥0.2. After applying the quality control parameters in the Excel output file, the total intensity of detected glycans of each sample was calculated. Finally, the relative abundance of each glycan was calculated by dividing the combined intensities for all detected charge states of each glycan by the total intensity of the sample.

#### Western blotting

After gel electrophoresis, Bio-Rad Mini-PROTEAN TGX gels (4–15%; Bio-Rad; Cat. No. 4561084) were rinsed with milliQ and transferred to Trans-Blot Turbo 0.2 μm Mini PVDF membranes (Bio-Rad; Cat. No. 170-4156). Blots were washed 3 times 5 min with 0.05% Tween in PBS (PBST) and blocked in 3% skim milk powder (Sigma; Cat. No. 70166) in PBST. Abs were incubated in blocking buffer for 1 h at room temperature or overnight at 4 °C. Primary Abs were incubated with the blots for 1–2 h at room temperature or overnight at 4 °C. Polyclonal antihuman IgG HRP Ab (1:2,500) was obtained from Dako/Agilent. Monoclonal antihuman CD22 HRP Ab (1:1,000) was obtained from Abcam (Cat. No Ab207727). Western blots were probed with Pierce ECL western blotting substrate (Thermo Fisher; Cat. No. 32106).

#### Flow cytometry

To stain Ramos B cells for BCR and CD22 expression, 200,000 cells were used for each condition. Cells were washed with washing buffer (PBA/PBS, 0.5% BSA, 0.02% NaN3) and incubated Fixation buffer (BioLegend, Cat. No. 420801) diluted 1:1 with washing buffer for 15–20 min. Cells were washed twice with washing buffer and incubated with a mix of Abs for 30 min in the dark. Cells were washed 3 times with wash buffer and taken up in wash buffer and stored at 4 °C until measuring time. Staining Abs used were anti-IgG Fc PE (Thermo Fisher; Cat. No. 12-4998-82) and anti-CD22 APC (BD Biosciences; Cat. No. 562860); isotype control Abs used were anti-CD3 PE (BD Biosciences; Cat No. 345765) and anti-Ig Kappa LC APC (BioLegend; Cat No. 316509).

**Abbreviations**
BCR: B-cell receptor
CDR: Complementarity Determining Region
FR: Framework
GBP: Glycan-Binding Partner
Ig: Immunoglobulin
IP: Immunoprecipitation
Lat-A -Latrunculin-A
LC: Liquid chromatography
mAb: Monoclonal antibody
MS: Mass spectrometry
NANA: N-Acetylneuraminic acid
NG: Non-glycosylated
PAL: Photoaffinity labeling
PLA: Proximity Ligation Assay
PV: Pervanadate
RA: Rheumatoid arthritis
SIGLEC: Sialic acid-binding, immunoglobulin-type lectin
SNA: *Sambucus Nigra* Agglutinin
ST6GAL: β-Galactoside α2–6 Sialyltransferase
UV: Ultraviolet
VDG: Variable domain glycan
WT: Wild type

## Author contributions

Miles D. Holborough-Kerkvliet (Conceptualization-Lead, Data curation-Lead, Formal analysis-Lead, Investigation-Lead, Methodology-Lead, Validation-Lead, Visualization-Lead, Writing—original draft-Lead, Writing—review & editing-Lead), Greta Mucignato (Investigation-Supporting), Sam J. Moons (Resources-Equal), Venetia Psomiadou (Resources-Equal), Rohit S.R. Konada (Resources-Equal), Nichole J. Pedowitz (Resources-Equal), Matthew R. Pratt (Resources-Equal), Theresa Kissel (Conceptualization-Supporting, Investigation-Supporting, Methodology-Supporting), Carolien A.M. Koeleman (Resources-Supporting), Rayman T.N. Tjokrodirijo (Formal analysis-Supporting, Resources-Supporting), Petrus A. van Veelen (Resources-Supporting), Thomas Huizinga (Project administration-Supporting, Supervision-Supporting), Karin A.J. van Schie (Conceptualization-Supporting, Investigation-Supporting, Methodology-Supporting, Supervision-Lead), Manfred Wuhrer (Resources-Supporting, Supervision-Supporting), Jennifer J. Kohler (Investigation-Supporting, Methodology-Supporting, Resources-Equal, Supervision-Supporting), Kimberly M. Bonger (Conceptualization-Supporting, Investigation-Supporting, Resources-Supporting, Supervision-Supporting), Thomas J. Boltje (Methodology-Lead, Resources-Lead, Supervision-Supporting), and Reinaldus E.M. Toes (Conceptualization-Lead, Funding acquisition-Lead, Project administration-Lead, Resources-Lead, Supervision-Lead, Writing—review & editing-Supporting).

## Supplementary Material

Supplmentary_Figures_cwad055

## Data Availability

The data underlying this article are available in the article and in its online supplementary material.
