# Supplementary material for: A photoaffinity glycan-labeling approach to investigate immunoglobulin glycan-binding partners
Source: Glycobiology. 2023 Jul 27;33(9):732–44. doi: 10.1093/glycob/cwad055 (PMC10627247; doi:10.1093/glycob/cwad055)
Supplement: Supplmentary_Figures_cwad055 [file supplmentary_figures_cwad055.pdf]

TITLE PAGE

SUPPLEMENTARY DATA

A photoaffinity glycan labeling approach to investigate immunoglobulin glycan binding partners

Keywords: Antibodies/B cell receptor/CD22/Photoaffinity labeling/Variable domain glycans

MD Holborough-Kerkvliet[a,g], G Mucignato[a], SJ Moons[b], V Psomiadou[b], RSK Konada[e], NJ Pedowitz[f], MR Pratt[f], T Kissel[a], CAM Koeleman[c], RTN Tjokrodirijo[c], PA van Veelen[c], T Huizinga[a], KAJ van Schie[a], M Wuhrer[c], JJ Kohler[e], KM Bonger[d], TJ Boltje[b], REM Toes[a]

a) Department of Rheumatology, Leiden University Medical Center, Albinusdreef 2, 2333 ZA, Leiden, The Netherlands

b) Department of Synthetic Organic Chemistry, Radboud University, Toernooiveld 1, Mercator III, 6525 ED, Nijmegen, The Netherlands

c) Center for Proteomics and Metabolomics, Leiden University Medical Center, Albinusdreef 2, 2333 ZA, Leiden, The Netherlands

d) Department of Synthetic Organic Chemistry, Radboud University, Heyendaalseweg 135, 6525 AJ, Nijmegen, The Netherlands

e) Department of Biochemistry, University of Texas Southwestern, 5323 Harry Hines Boulevard, Dallas, TX 75390-09185

f) Department of Chemistry, University of Southern California, Los Angeles, California 90089, United States

g) Corresponding author: M.D.Holborough-Kerkvliet@LUMC.nl

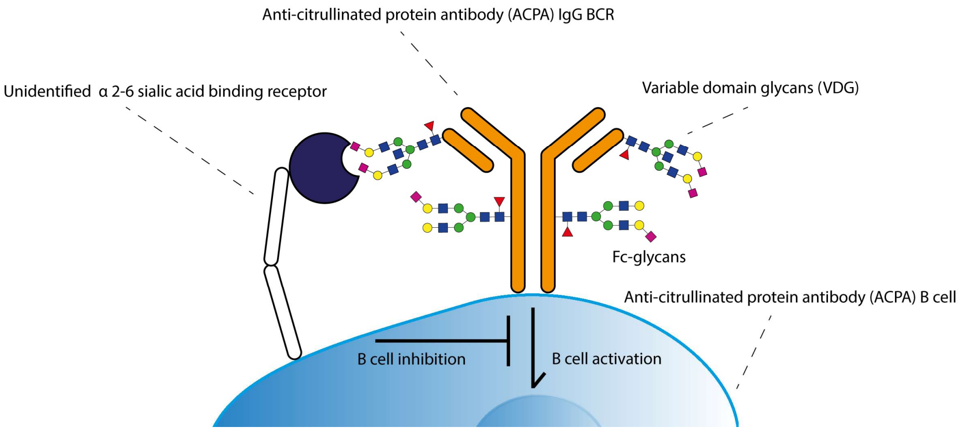

Supplementary Figure 1

Graphical depiction of our proposed hypothesis: ACPA B cell receptors that carry α2-6-linked sialic acids could engage (immunomodulatory) sialic acid binding receptors in *cis*[a].

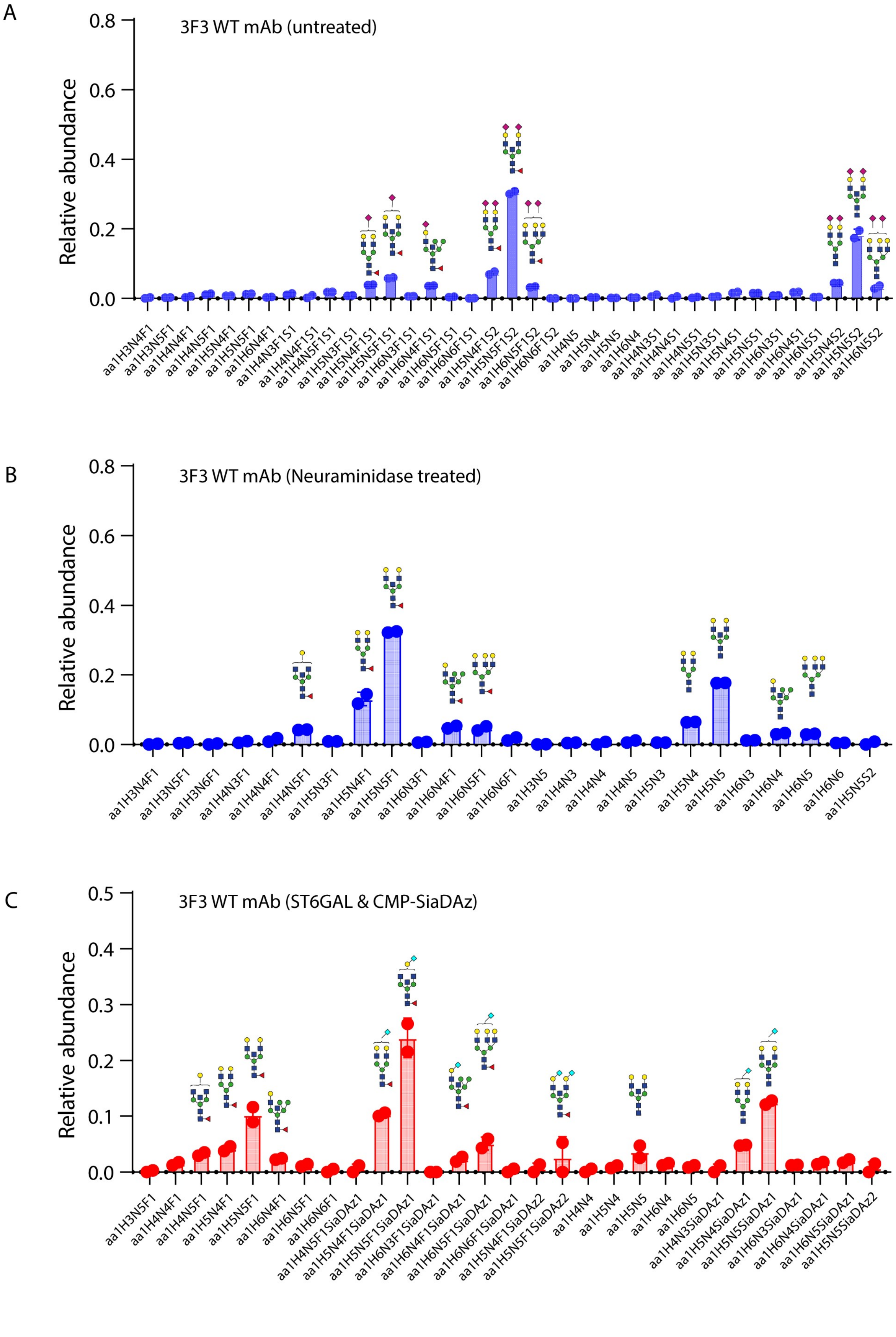
A
3F3 WT mAb (untreated)
Relative abundance
0.8
0.6
0.4
0.2
0.0
aa1H3N4F1
aa1H3N5F1
aa1H4N4F1
aa1H4N5F1
aa1H5N4F1
aa1H5N5F1
aa1H6N4F1
aa1H4N3F1S1
aa1H4N4F1S1
aa1H4N5F1S1
aa1H5N3F1S1
aa1H5N4F1S1
aa1H5N5F1S1
aa1H6N3F1S1
aa1H6N4F1S1
aa1H6N5F1S1
aa1H6N6F1S1
aa1H5N4F1S2
aa1H5N5F1S2
aa1H6N5F1S2
aa1H6N6F1S2
aa1H4N5
aa1H5N4
aa1H5N5
aa1H6N4
aa1H4N3S1
aa1H4N4S1
aa1H4N5S1
aa1H5N3S1
aa1H5N4S1
aa1H5N5S1
aa1H6N3S1
aa1H6N4S1
aa1H6N5S1
aa1H5N4S2
aa1H5N5S2
aa1H6N5S2
B
3F3 WT mAb (Neuraminidase treated)
Relative abundance
0.8
0.6
0.4
0.2
0.0
aa1H3N4F1
aa1H3N5F1
aa1H3N6F1
aa1H4N3F1
aa1H4N4F1
aa1H4N5F1
aa1H5N3F1
aa1H5N4F1
aa1H5N5F1
aa1H6N3F1
aa1H6N4F1
aa1H6N5F1
aa1H6N6F1
aa1H3N5
aa1H4N3
aa1H4N4
aa1H4N5
aa1H5N3
aa1H5N4
aa1H5N5
aa1H6N3
aa1H6N4
aa1H6N5
aa1H6N6
aa1H5N5S2
C
3F3 WT mAb (ST6GAL & CMP-SiaDAz)
Relative abundance
0.5
0.4
0.3
0.2
0.1
0.0
aa1H3N5F1
aa1H4N4F1
aa1H4N5F1
aa1H5N4F1
aa1H5N5F1
aa1H6N4F1
aa1H6N5F1
aa1H6N6F1
aa1H4N5F1SiaDAz1
aa1H5N4F1SiaDAz1
aa1H5N5F1SiaDAz1
aa1H6N3F1SiaDAz1
aa1H6N4F1SiaDAz1
aa1H6N5F1SiaDAz1
aa1H6N6F1SiaDAz1
aa1H5N4F1SiaDAz2
aa1H5N5F1SiaDAz2
aa1H4N4
aa1H5N4
aa1H5N5
aa1H6N4
aa1H6N5
aa1H4N3SiaDAz1
aa1H5N4SiaDAz1
aa1H5N5SiaDAz1
aa1H6N3SiaDAz1
aa1H6N4SiaDAz1
aa1H6N5SiaDAz1
aa1H5N5SiaDAz2

Supplementary Figure 2

A) Relative abundances of the relative abundance of the enzymatically released complex and hybrid type Fc and variable domain glycans of a ACPA (3F3 WT) monoclonal antibody produced in HEK cells that passed quality control parameters. Abundant glycans are additionally annotated graphically. N=2 experimental replicates.

B) Relative abundances of the relative abundance of glycans of the 3F3 WT monoclonal antibody shown in Supplementary Figure 2A treated with sialidase. Abundant glycans are additionally annotated graphically. N=2 experimental replicates.

C) Relative abundances of the relative abundance of the 3F3 WT monoclonal antibody of Supplementary Figure 2A treated with sialidase and exo-enzymatically engineered with ST6GAL and CMP-SiaDAz. Abundant glycans are additionally annotated graphically. N=2 experimental replicates.

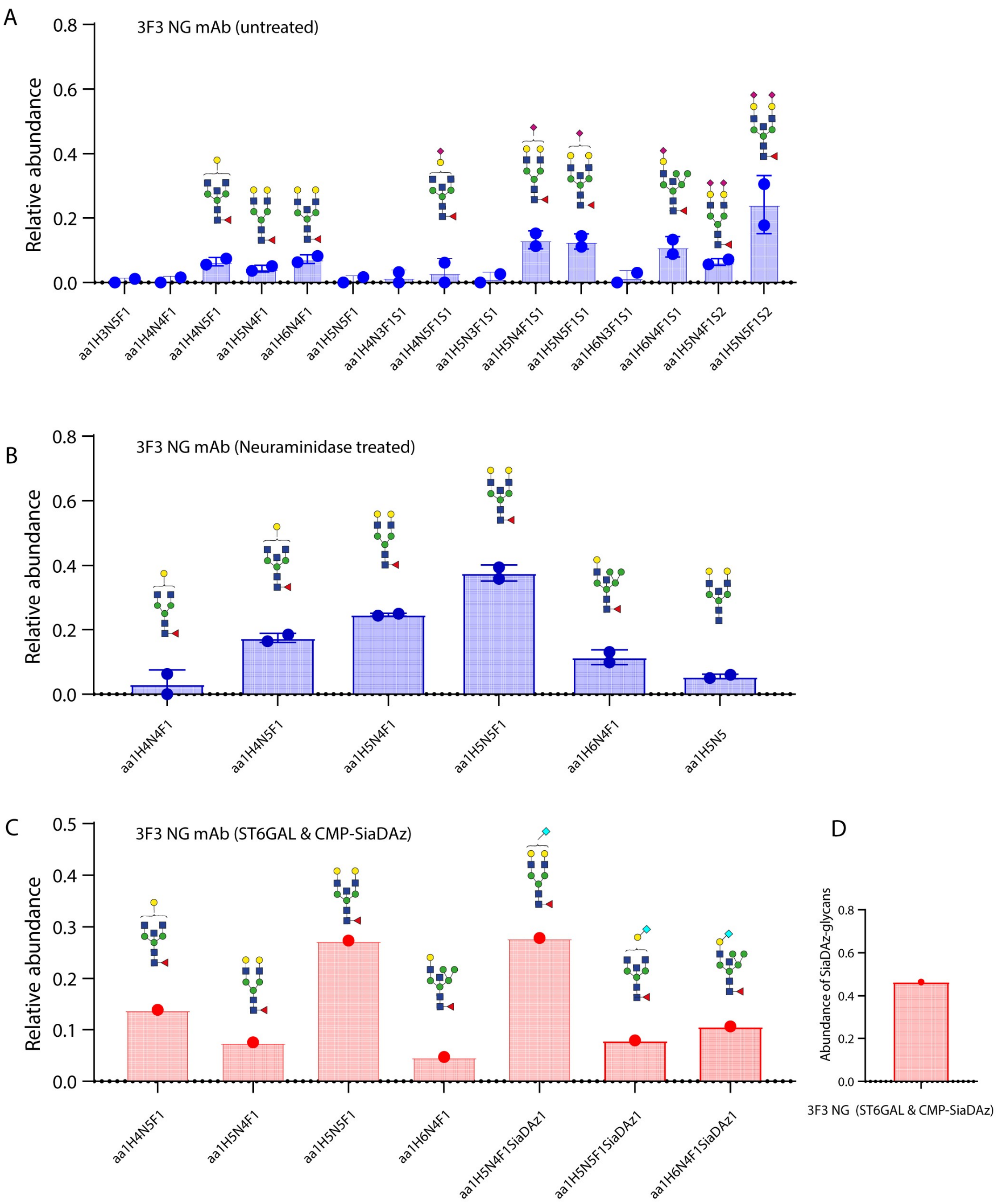
A
3F3 NG mAb (untreated)
Relative abundance
0.0
0.2
0.4
0.6
0.8
aa1H3N5F1
aa1H4N4F1
aa1H4N5F1
aa1H5N4F1
aa1H6N4F1
aa1H5N5F1
aa1H4N3F1S1
aa1H4N5F1S1
aa1H5N3F1S1
aa1H5N4F1S1
aa1H5N5F1S1
aa1H6N3F1S1
aa1H6N4F1S1
aa1H5N4F1S2
aa1H5N5F1S2
B
3F3 NG mAb (Neuraminidase treated)
Relative abundance
aa1H4N4F1
aa1H4N5F1
aa1H5N4F1
aa1H5N5F1
aa1H6N4F1
aa1H5N5
C
3F3 NG mAb (ST6GAL & CMP-SiaDAz)
Relative abundance
0.0
0.1
0.2
0.3
0.4
0.5
aa1H4N5F1
aa1H5N4F1
aa1H5N5F1
aa1H6N4F1
aa1H5N4F1SiaDAz1
aa1H5N5F1SiaDAz1
aa1H6N4F1SiaDAz1
D
Abundance of SiaDAz-glycans
3F3 NG (ST6GAL & CMP-SiaDAz)

Supplementary Figure 3

A) Relative abundances of the enzymatically released complex and hybrid type Fc glycans of a ACPA (3F3 NG) monoclonal antibody produced in HEK cells that passed quality control parameters. Abundant glycans are additionally annotated graphically. N=2 experimental replicates.

B) Relative abundances of glycans of the 3F3 NG monoclonal antibody shown in Supplementary Figure 3A treated with sialidase. N=2 experimental replicates.

C) Relative abundances of the 3F3 NG monoclonal antibody of Supplementary Figure 3A treated with sialidase and exo-enzymatically engineered with ST6GAL and CMP-SiaDAz. N=1 experimental replicates.

D) Relative abundances of all detected complex- and hybrid-type glycans that carry a SiaDAz-molecule. N=1 experimental replicates.

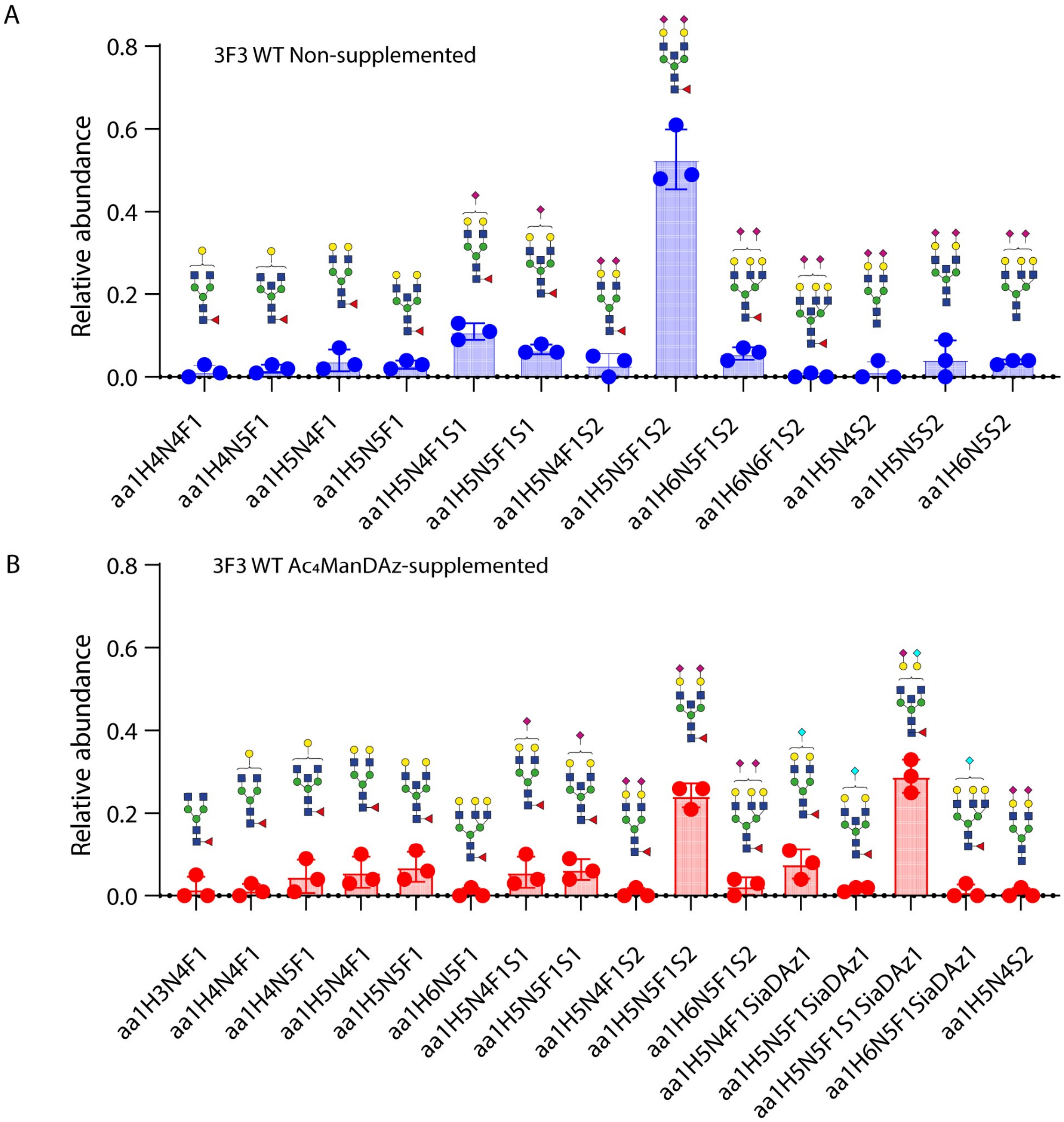

Supplementary Figure 4

A) Relative abundances of the enzymatically released complex and hybrid type Fc and variable domain glycans of ACPA (3F3 WT) BCRs that passed quality control parameters and were obtained from cells cultured without Ac_4MaNDAz.

B) Relative abundances of the enzymatically released complex and hybrid type Fc and variable domain glycans of ACPA (3F3 WT) BCRs that passed quality control parameters and were obtained from cells cultured with Ac_4MaNDAz (100 µM, 72 hours).

A

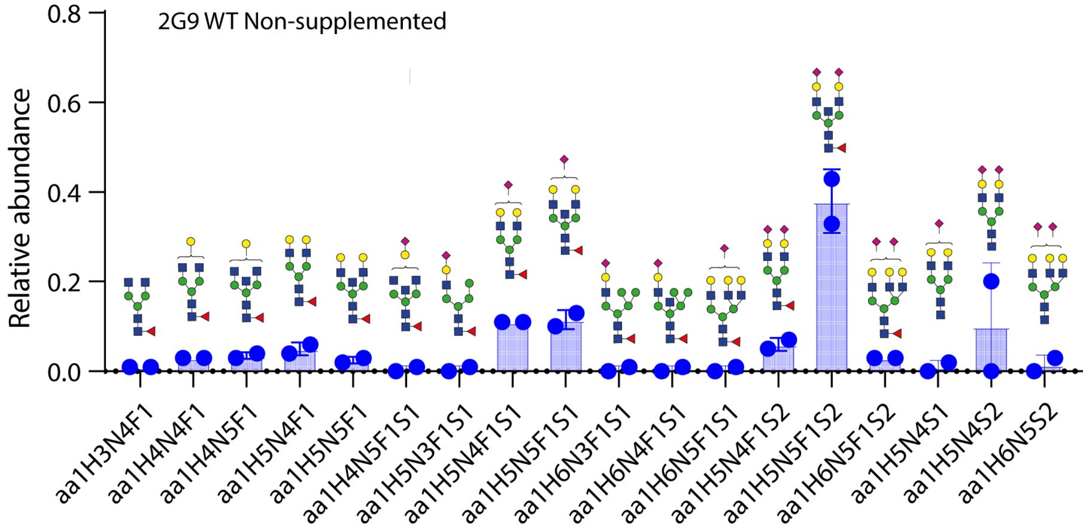

B

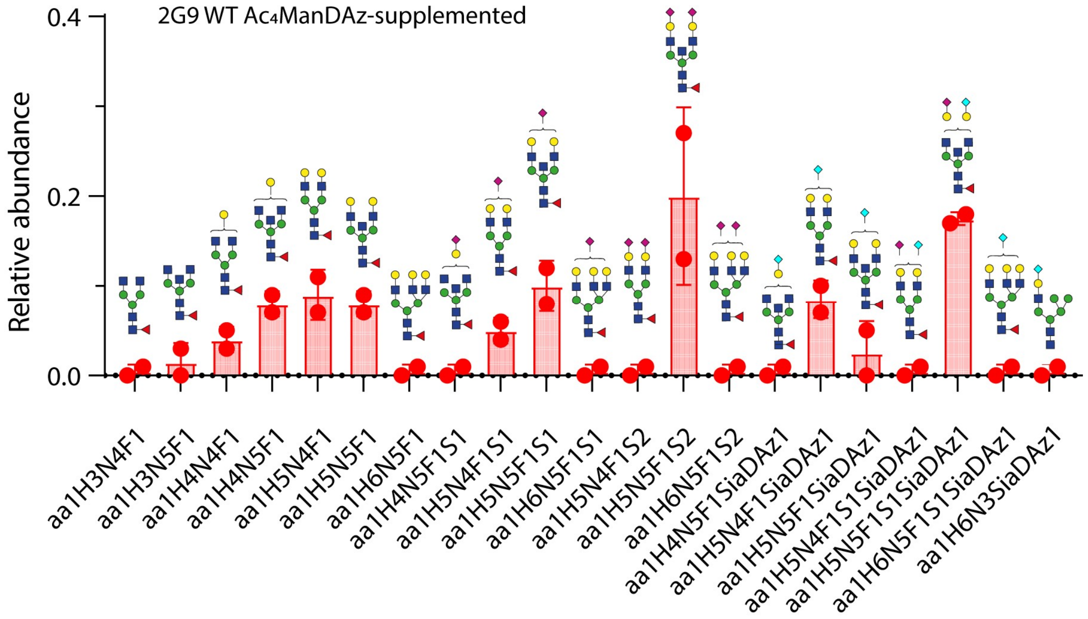

Supplementary Figure 5

A) Relative abundances of the enzymatically released complex and hybrid type Fc and variable domain glycans of ACPA (2G9 WT) BCRs that passed quality control parameters and were obtained from cells cultured without $Ac_4MaNDAz$.

B) Relative abundances of the enzymatically released complex and hybrid type Fc and variable domain glycans of ACPA (2G9 WT) BCRs that passed quality control parameters and were obtained from cells cultured with $Ac_4MaNDAz$ (100 µM, 72 hours).

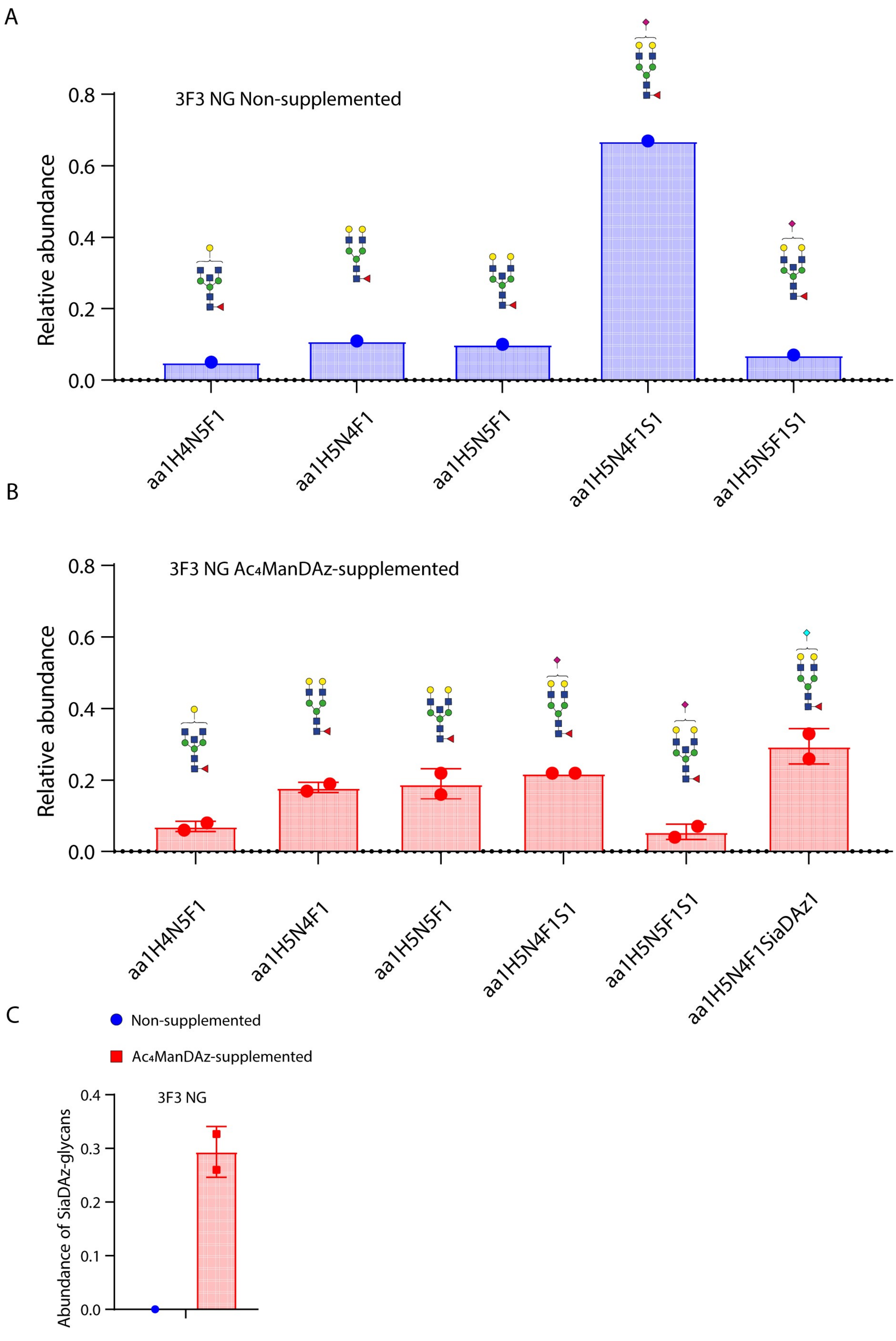
A
3F3 NG Non-supplemented
Relative abundance
0.0
0.2
0.4
0.6
0.8
aa1H4N5F1
aa1H5N4F1
aa1H5N5F1
aa1H5N4F1S1
aa1H5N5F1S1
B
3F3 NG Ac4ManDAz-supplemented
Relative abundance
0.0
0.2
0.4
0.6
0.8
aa1H4N5F1
aa1H5N4F1
aa1H5N5F1
aa1H5N4F1S1
aa1H5N5F1S1
aa1H5N4F1SiaDAz1
C
Non-supplemented
Ac4ManDAz-supplemented
3F3 NG
Abundance of SiaDAz-glycans
0.0
0.1
0.2
0.3
0.4

Supplementary Figure 6

A) Relative abundances of the enzymatically released complex and hybrid type Fc glycans of ACPA (3F3 NG) BCRs that passed quality control parameters and were obtained from cells cultured without Ac_4MaNDAz. N=1 experimental replicates.

B) Relative abundances of the enzymatically released complex and hybrid type Fc glycans of ACPA (3F3 NG) BCRs that passed quality control parameters and were obtained from cells cultured with Ac_4MaNDAz (100 µM, 72 hours). N=2 experimental replicates.

C) Relative abundances of all detected complex and hybrid type glycans that carry a SiaDAz-molecule. N=1 and 2 experimental replicates for 3F3 NG non-supplemented and 3F3 NG Ac_4MaNDAz-supplemented, respectively.

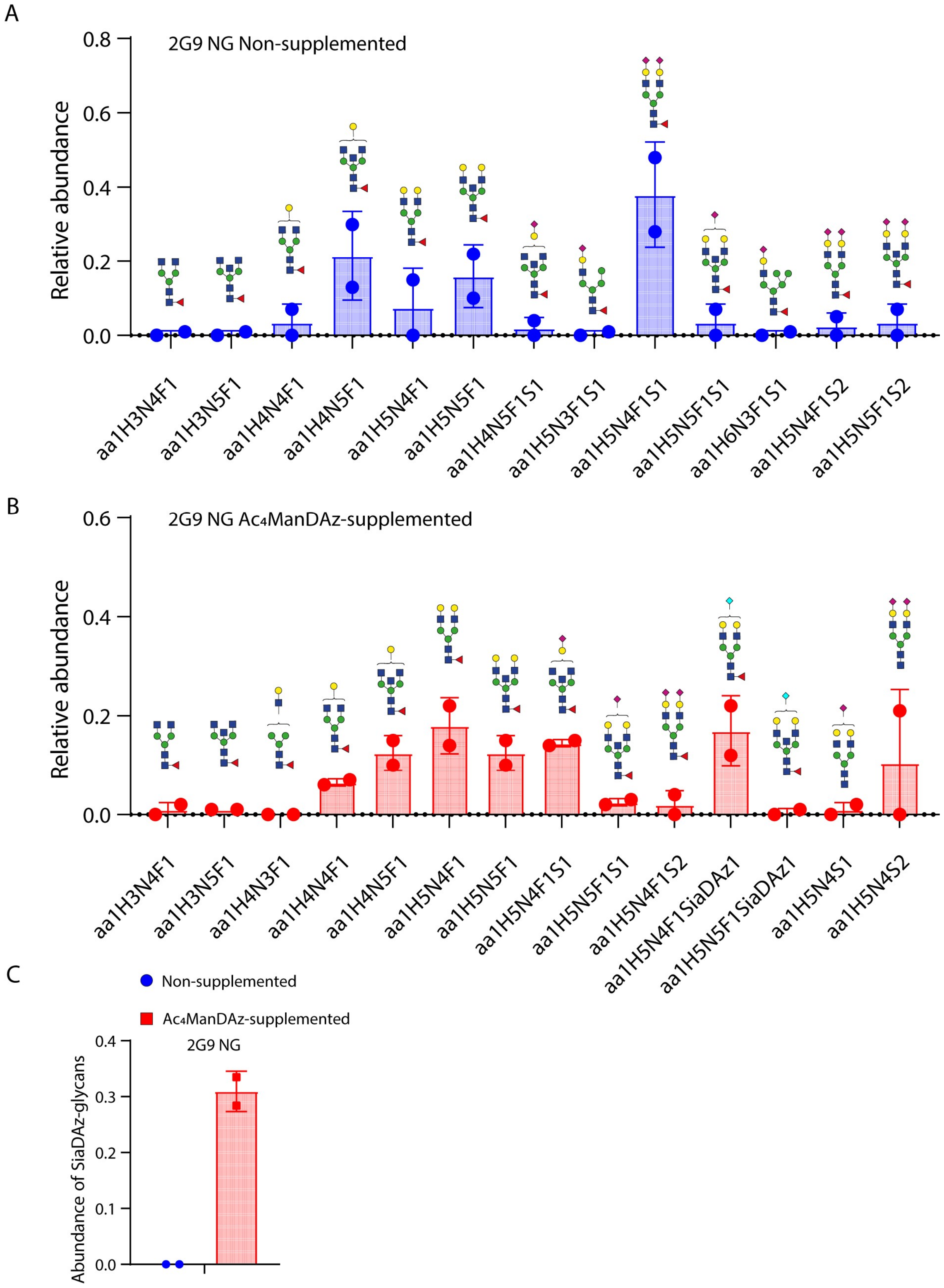
A
2G9 NG Non-supplemented
Relative abundance
0.8
0.6
0.4
0.2
0.0
aa1H3N4F1
aa1H3N5F1
aa1H4N4F1
aa1H4N5F1
aa1H5N4F1
aa1H5N5F1
aa1H4N5F1S1
aa1H5N3F1S1
aa1H5N4F1S1
aa1H5N5F1S1
aa1H6N3F1S1
aa1H5N4F1S2
aa1H5N5F1S2
B
2G9 NG Ac4ManDAz-supplemented
Relative abundance
0.6
0.4
0.2
0.0
aa1H3N4F1
aa1H3N5F1
aa1H4N3F1
aa1H4N4F1
aa1H4N5F1
aa1H5N4F1
aa1H5N5F1
aa1H5N4F1S1
aa1H5N5F1S1
aa1H5N4F1S2
aa1H5N4F1SiaDAz1
aa1H5N5F1SiaDAz1
aa1H5N4S1
aa1H5N4S2
C
Non-supplemented
Ac4ManDAz-supplemented
2G9 NG
Abundance of SiaDAz-glycans
0.4
0.3
0.2
0.1
0.0

Supplementary Figure 7

A) Relative abundances of the enzymatically released complex and hybrid type Fc glycans of ACPA (2G9 NG) BCRs that passed quality control parameters and were obtained from cells cultured without Ac_4MaNDAz.

B) Relative abundances of the enzymatically released complex and hybrid type Fc glycans of ACPA (2G9 NG) BCRs that passed quality control parameters and were obtained from cells cultured with Ac_4MaNDAz (100 µM, 72 hours).

C) Relative abundances of all detected complex and hybrid type glycans that carry a SiaDAz-molecule. N=2 experimental replicates for 2G9 NG non-supplemented and 2G9 NG Ac_4MaNDAz-supplemented.

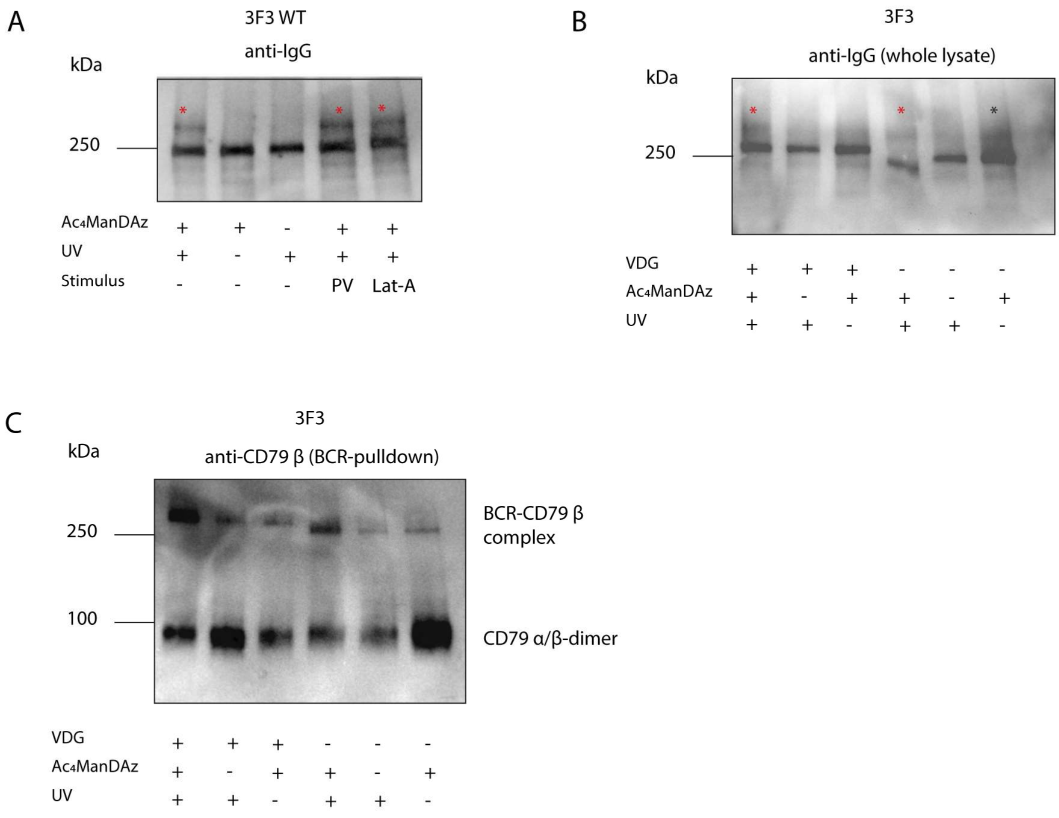

Supplementary Figure 8

A) Anti-IgG western blots showing the presence of UV and Ac_4MaNDAz-dependent higher molecular weight bands in lysates of unstimulated cells or cells stimulated with pervanadate (PV) or latrunculin A (Lat-A).

B) Anti-IgG western blots showing the presence of UV and Ac_4MaNDAz-dependent higher molecular weight bands in both WT and NG samples. UV and Ac_4MaNDAz-dependent complexes are annotated with a red asterisk. Higher molecular weight bands not dependent on UV and Ac_4MaNDAz are annotated with a black asterisk.

C) Anti-CD79 β western blot after pulldown of the BCR. CD79 β can be found at the corresponding height of higher molecular weight bands observed in anti-IgG blots. The CD79 α/β dimer can be seen at its expected height, between 77 and 100 kDa.

Table SI

	Sequence CD79 β	#PSMs		Sequence CD79 α	#PSMs
#1 3F3 WT + UV + M	QEMDENPQQLK	3			
	IWQSPR	3			
#1 - 3F3 WT + UV - M	NA	NA			
#1 - 3F3 WT (S10) + UV + M	QEMDENPQQLK	3		SHGGIYVCR	3
	IWQSPR	3			
	WSVGEHPGQE	3			
#1 - 3F3 WT (S10) + UV - M	NA	NA			
#1 - MDL-KO	NA	NA			
#2 - 3F3 WT + UV + M	VMGFSTLAQLK	1			
#2 - 3F3 WT + UV - M	NA	NA			
#2 - 3F3 WT (S10) + UV + M	NA (was expected but not found)	NA (was expected but not found)			
#2 - 3F3 WT (S10) + UV - M	NA	NA			
#2 - 3F3 NG + UV + M	QEMDENPQQLK	1			
#2 - 3F3 NG + UV - M	NA	NA			
#2 - MDL-KO	NA	NA			

Table showing identified CD79 α and β-derived peptides after the tryptic digest of the observed higher molecular weight bands observed above the BCR in the +UV, +Ac_4ManDAz-conditions and the respective negative control bands.

FOOTNOTES

A. All canonical monosaccharide symbols in this manuscript follow the SNFG (Symbol Nomenclature for Glycans) system (Varki *et al.* 2015). The non-canonical, synthetic sialic acid, SiaDAz is annotated as a cyan diamond.
